# Probabilistic prediction of rock avalanche runout using a numerical model

**DOI:** 10.1007/s10346-022-01939-y

**Published:** 2022-08-15

**Authors:** Jordan Aaron, Scott McDougall, Julia Kowalski, Andrew Mitchell, Natalia Nolde

**Affiliations:** 1 Geological Institute, ETH Zürich, Zurich, Switzerland; 2 Now at Swiss Federal Institute for Forest, Snow and Landscape Research WSL, Birmensdorf, Switzerland; 3 Department of Earth, Ocean and Atmospheric Sciences, The University of British Columbia, Vancouver, Canada; 4 Computational Geoscience, University of Göttingen, Göttingen, Germany; 5 Now at Methods for Model-Based Development in Computational Engineering, RWTH Aachen University, Aachen, Germany; 6 Department of Statistics, The University of British Columbia, Vancouver, Canada; 7 BGC Engineering Inc., Vancouver, Canada

**Keywords:** Rock avalanches, Probabilistic prediction, Runout modelling, Probabilistic calibration

## Abstract

Rock avalanches can be a significant hazard to communities located in mountainous areas. Probabilistic predictions of the 3D impact area of these events are crucial for assessing rock avalanche risk. Semi-empirical, calibration-based numerical runout models are one tool that can be used to make these predictions. When doing so, uncertainties resulting from both noisy calibration data and uncertain governing movement mechanism(s) must be accounted for. In this paper, a back-analysis of a database of 31 rock avalanche case histories is used to assess both of these sources of uncertainty. It is found that forecasting results are dominated by uncertainties associated with the bulk basal resistance of the path material. A method to account for both calibration and mechanistic uncertainty is provided, and this method is evaluated using pseudo-forecasts of two case histories. These pseudo-forecasts show that inclusion of expert judgement when assessing the bulk basal resistance along the path can reduce mechanistic uncertainty and result in more precise predictions of rock avalanche runout.

**Supplementary Information:**

The online version contains supplementary material available at 10.1007/s10346-022-01939-y.

## Introduction

Rock avalanches are extremely rapid flows of fragmented rock (Hungr et al. 2014). Predicting the potential impact area of a rock avalanche before it initiates is a crucial step in the risk analysis of these landslides. The recent West Salt Creek (White et al. 2015; Coe et al. 2016) and Mt. Meager (Guthrie et al. 2012) events highlight the need for accurate forecasts of rock avalanche motion, as in these two cases the surprisingly long runout of the events caused fatalities, and the formation of a potentially hazardous landslide dam, respectively. Additionally, Coe et al. (2018) suggest that climate warming may lead to increased frequency of rock avalanche occurrence, which in combination with increasing development pressures may increase demand for these sorts of predictions in the near future.

**Fig. 1:**
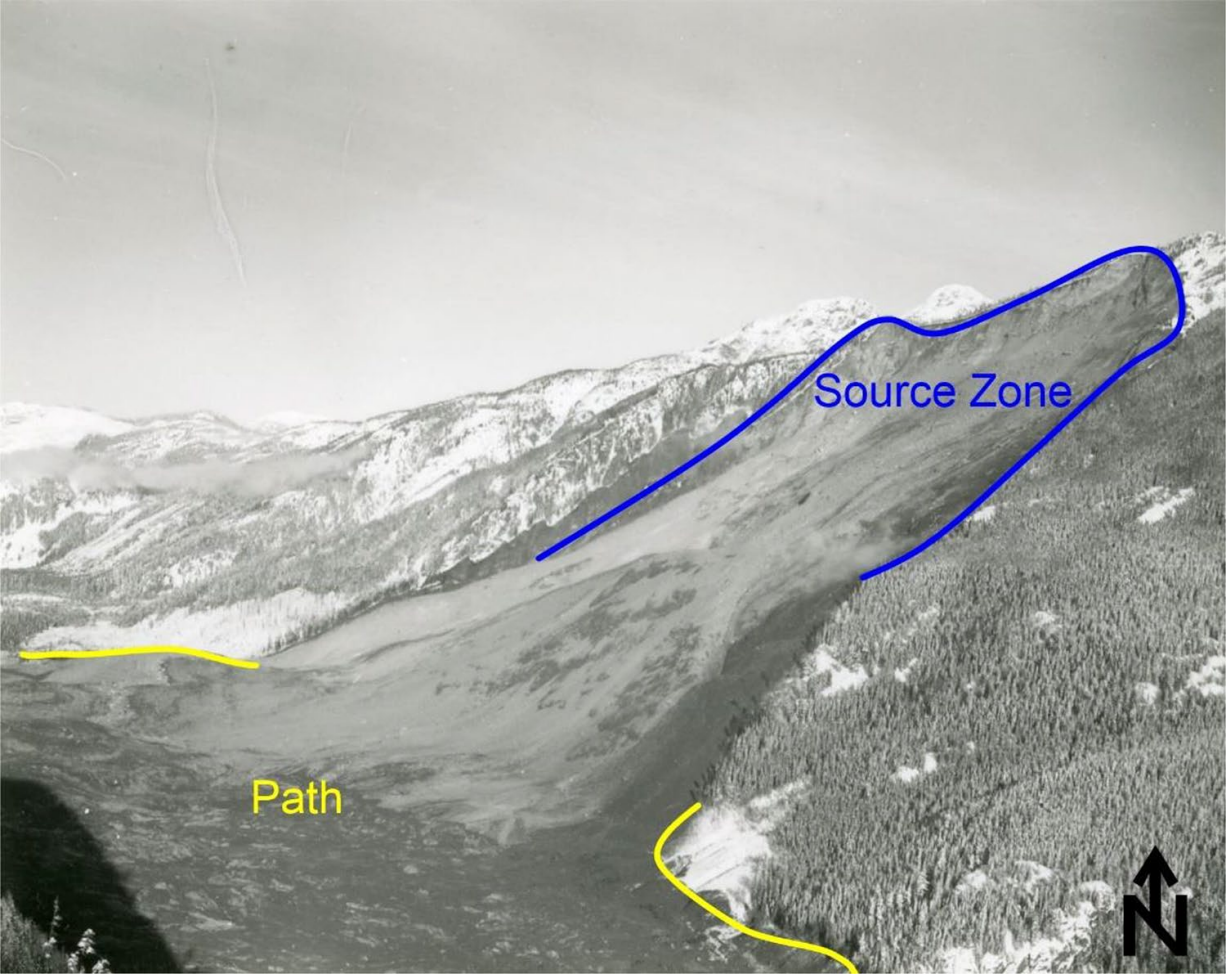
Example rock avalanche showing source zone (blue), and path (red). Hope Slide photo courtesy British Columbia Ministry of Transportation and Infrastructure

As shown on Fig. 1, rock avalanches initiate as large rock slope failures on steep mountain slopes (Hungr et al. 2014). The failed rock mass initially accelerates in the source zone (Fig. 1), where shearing is typically localized along a basal rupture plane, and the movement of the landslide mass can remain coherent (De Blasio and Crosta 2013). As the accelerating mass vacates the source zone, it progressively disintegrates and turns flow-like (De Blasio 2011; De Blasio and Crosta 2013; Aaron and Hungr 2016b). Following fragmentation, the mass can behave as a frictional fluid, and basal shearing occurs between the flowing fragments of rock and the material that is being overridden (path on Fig. 1), which can include material such as glacier ice, bedrock, and/or saturated sediments (e.g. Hungr and Evans 2004; Sosio et al. 2008; Dufresne et al. 2019). Finally, the fragmented mass comes to rest and forms a deposit.

Beginning with the work of Heim (1932), many researchers have noted an apparent increase in rock avalanche mobility with increasing volume (e.g., Heim 1932; Scheidegger 1973; Li 1983; Whittall et al. 2017). The reasons for this observation remain controversial, making forecasting rock avalanche runout a uniquely challenging task (e.g., Davies et al. 1999; Legros 2002; Hungr and Evans 2004; Johnson et al. 2016; Manzanal et al. 2016; Aaron and McDougall 2019). Since no consensus has emerged regarding the macro-scale movement mechanisms that govern rock avalanche motion, constructing precise numerical models based on fundamental material properties is difficult. However, there is a need for methods to predict the motion of these flows within the landslide risk analysis framework (e.g., Hungr 2016). It is the authors’ opinion that a combination of empirical tools and semi-empirical numerical models (e.g., Hungr 1995) are the most promising methods for making practical predictions at the moment.

Empirical relationships derived from observations of past landslides are well-suited for screening-level runout analysis. These methods are typically based on correlations between variables such as landslide volume, confinement conditions, fall height, runout length, and deposit area (e.g., Li 1983; Iverson et al. 1998; Griswold and Iverson 2008; Mitchell et al. 2020b). These correlations result in tools that are easy to use yet powerful, as predictions can be made within a probabilistic framework (e.g., Hungr et al. 2005; Griswold and Iverson 2008; Mitchell et al. 2020b). However, the resulting wide confidence bands, due in part to small sample sizes of historical datasets, as well as their limited ability to provide landslide intensity estimates (e.g., flow depth, velocity and impact pressures), generally limit their use to high-level hazard mapping and risk assessment applications.

Advanced numerical models are typically used to provide more detailed estimates of impact area and intensity parameters. Many models that can simulate landslide runout across three-dimensional terrain have been proposed, and recent summaries are provided by McDougall (2017) and Ho et al. (2018). These types of models are capable of simulating complex internal stress distributions (Savage and Hutter 1989), entrainment (e.g., McDougall and Hungr 2005; Cuomo et al. 2014; Iverson and Ouyang 2015), and the initial coherence exhibited by rock avalanches (Aaron and Hungr 2016b). So far, these models have been used extensively for back-analysis; however, a methodology for selecting parameters for probabilistic forward analysis of rock avalanches is still lacking, despite recent progress which explore probabilistic methods for parameter selection and scenario evaluation, as well as the transferability and sensitivity of calibrated parameters (Quan Luna 2012; Pirulli 2016; Mergili et al. 2018a, b; Sun et al. 2020, 2021). Progress has also been made in advanced methods for quantifying model sensitivities generally (e.g., Asheghi et al. 2020; Razavi et al. 2021); however, simplified tools for forward predictions are still required in practice.

One approach to forecast runout with advanced numerical models is to select parameter values based on the results of back-analyses of similar rock avalanches. This semi-empirical approach, termed the “equivalent fluid” principle by Hungr (1995), forms the basis for the model calibration and forecasting methodologies described in the present work. These forecasts must account for uncertainties arising from both inherent statistical noise, as well as a lack of knowledge of the phenomenon, analogous to aleatoric and epistemic uncertainty, respectively (Bedi and Harrison 2013). Within the equivalent fluid framework, these two types of uncertainties manifest as:

1. Uncertainty inherent in back-analysis (e.g., Fischer et al. 2015). This includes estimation uncertainty when fitting the model, as well as the numerical model being an imperfect representation of reality (Beven 2005; Doherty and Welter 2010; Aaron et al. 2019). In this paper, we refer to this as “calibration uncertainty,” and it is the primary source of aleatoric uncertainty in the analyzed forecasting problem.
2. Uncertainty in evaluating how similar a future rock avalanche event will be to previously back-analyzed cases. This type of uncertainty primarily results from uncertainties in the mechanism(s) that will govern the motion of a potential rock avalanche. In this paper, we refer to this as “mechanistic uncertainty,” and it represents the majority of epistemic uncertainty when predicting rock avalanche motion.

This paper details a methodology for making predictions of rock avalanche motion, which can be applied in practice. Recent work has focused on developing methodologies for back-analysis of single case histories (e.g., Fischer et al. 2015); however, the implementation and testing of a formal method for combining back-analysis results from multiple case histories to make probabilistic predictions are still lacking. Here, we implement and test such a methodology. The new methodology fits naturally into the landslide risk analysis framework and accounts for both calibration and mechanistic uncertainty, described above.

Firstly, in the “Data” section, we describe a database of 31 rock avalanche case histories that is used in this work to investigate the sources of calibration and mechanistic uncertainty. Secondly, in the “Quantification of calibration uncertainty” section, we describe a probabilistic calibration methodology and apply it to back-analyze the database. Thirdly, in the “Assessment of mechanistic uncertainty” section, we provide a methodology to assess the similarity of a potential future event to the thirty-one case histories assembled in the database. Finally, in the “Evaluation of forecasting methodology” section, we test this new framework using pseudo-forecasts of two rock avalanche case histories and compare these forecasts to those made by an empirical-statistical tool. We conclude by discussing the strengths and weaknesses of the presented approach, and highlighting opportunities for future work.

## Data

**Fig. 2:**
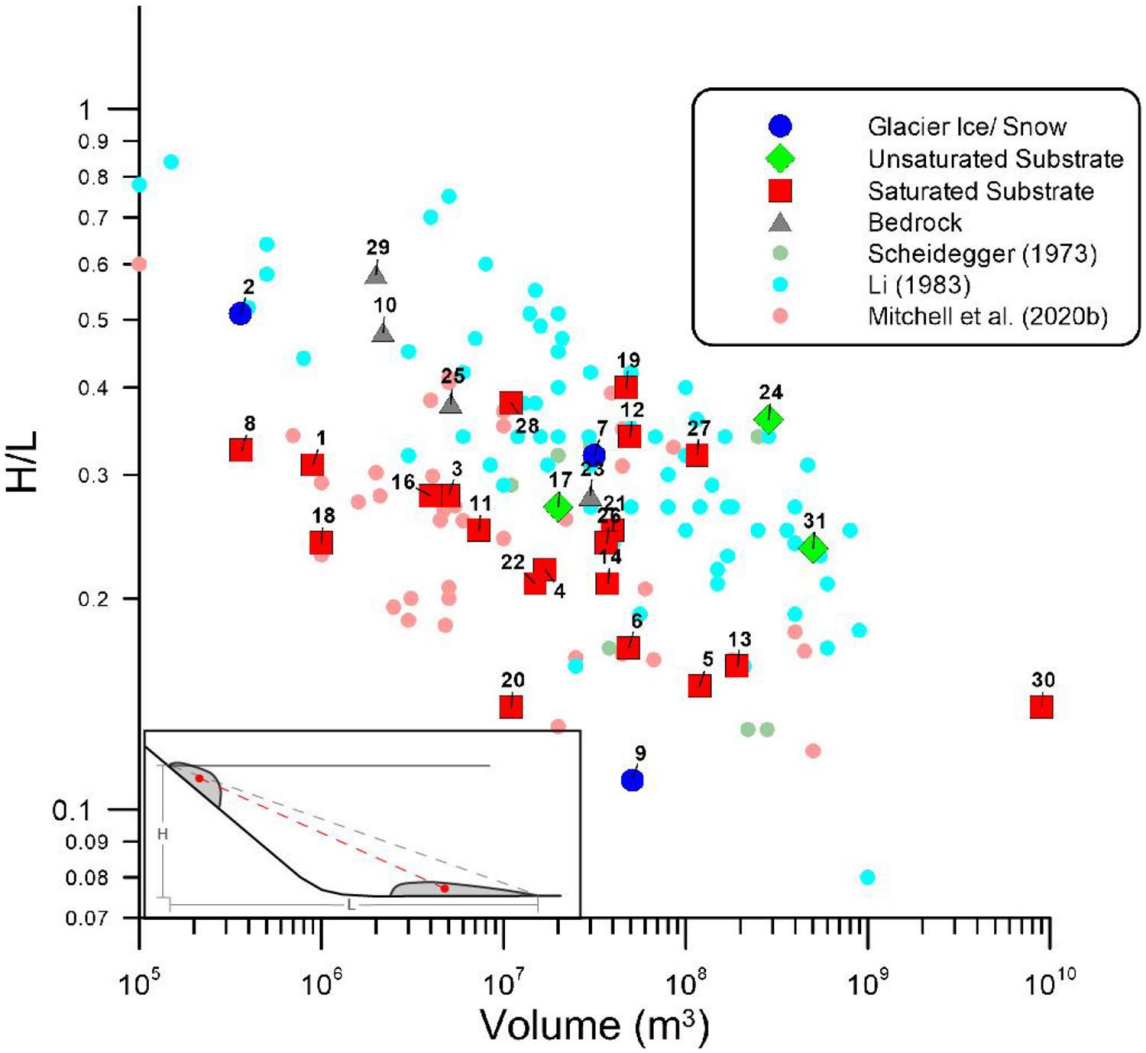
Volume vs. H/L of cases in the database of rock avalanche case histories. For comparison, H/L data collected by Scheidegger (1973), Li (1983), and Mitchell et al. (2020b) are shown. Cases are sorted by path material. References to DanW and/or Dan3D analyses, as well as papers summarizing the case histories, are provided in Supplementary Table S1. Labels: 1. Zymoetz, 2. Crammont, 3. Six des Eaux, 4. Huascaran, 5. Kolka, 6. Mt. Meager, 7. Mt. Steele, 8. Nomash River, 9. Sherman Glacier, 10. Thurweiser, 11. McAuley Creek, 12. Val Pola, 13. Avalanche Lake, 14. Goldau, 15. Mystery Creek, 16. Turnoff Creek, 17. Madison Canyon, 18. Chisca, 19. Hope, 20. West Salt Creek, 21. Frank, 22. Guinsaugon, 23. Bingham Canyon, 24. Sentinel, 25. Daubensee, 26. Rinderhorn, 27. Rautispitz, 28. Platten, 29. Chehalis, 30. Flims, and 31. Molveno. Inset: explanation of the H/L ratio, with H defined as the vertical height between the tip of the scarp and toe of the deposit, and L the horizontal distance between the two

A database of 31 rock avalanche case histories has been assembled as part of this work. A detailed summary of the database is provided in the Supplementary Information. Figure 2 shows the volume vs. fall height over runout length (H/L) relationship of the cases in the rock avalanche database used to develop the proposed probabilistic runout forecasting framework. The H/L ratio is explained in the Fig. 2 inset. With the exception of the Bingham Canyon rock avalanche, all cases in the database are natural rock avalanches. A detailed description of the Bingham Canyon rock avalanche is provided in (Moore et al. 2017). The cases span a wide range of mobility, from the “expected” value for dry fragmented debris of 0.6 (Hsu 1975) to the Sherman Glacier rock avalanche (H/L of 0.1). Figure 2 also shows the likely material overrun by the analyzed rock avalanches once they vacated the source zone. Most cases in the database likely overran saturated substrate; however, cases that overran bedrock, glacial ice, and likely unsaturated sediments were also back-analyzed.

To the authors’ knowledge, this database is the largest collection of rock avalanches assembled and analyzed with a consistent calibration methodology using a three-dimensional runout model (as described below). However, we acknowledge that a sample size of 31 cases is a limited sample of rock avalanche behavior. This will introduce uncertainty into the resulting predictions, which will be explored when evaluating the proposed forecasting methodology. However, the expected bias of the assembled dataset can also be assessed by comparing the analyzed case histories with the larger databases of mobility estimates shown in Fig. 2. Figure 2 also shows H/L values measured by Scheidegger (1973), Li (1983) and the Canadian database that underlies PRE-RA (Mitchell et al. 2020b), an empirical runout estimation tool that is described in more detail below. For cases that are duplicated in the datasets, the most recent reference is used. Comparing the data, on average, the cases analyzed in the present work appear more mobile than those compiled by Scheidegger (1973) and Li (1983), and similar to those compiled by Mitchell et al. (2020b). It can thus be expected that the resulting forecasts may be biased towards high moblity. This can be partially accounted for using expert judgement, as detailed below; however, this will be an important factor when interpreting resulting forecasts.

## Quantification of calibration uncertainty

### Probabilistic calibration methodology

The database of rock avalanche case histories has been back-analyzed with a numerical model, using a probabilistic methodology that quantifies calibration uncertainty. In this work, we use the runout model Dan3D-Flex (Aaron and Hungr 2016b), which combines a solid mechanics based flexible block model with Dan3D, a fluid-mechanics based runout model. In Dan3D-Flex, the mass is initially simulated as a flexible block that can translate and rotate over 3D topography. At a user specified time, the mass turns “flow-like,” and its behavior is governed by the Dan3D simulation algorithm (McDougall and Hungr 2004). The Dan3D algorithm is a depth averaged, Lagrangian description of the equations of motion, solved using smooth particle hydrodynamics (McDougall and Hungr 2004; Hungr and McDougall 2009). A key feature of Dan3D is that it simulates the development of strain-dependent internal stresses, whose magnitude is governed by an internal friction angle (Savage and Hutter 1989; Hungr 2008). Dan3D-Flex is thus suitable to simulate cases that fail along a planar rupture surface, such as a bedding plane, as flow-like behavior only occurs sometime after the onset of motion (as described in the introduction). Although we use Dan3D-Flex here, the probabilistic simulation methodology we use is generally applicable to other numerical runout models.

**Fig. 3:**
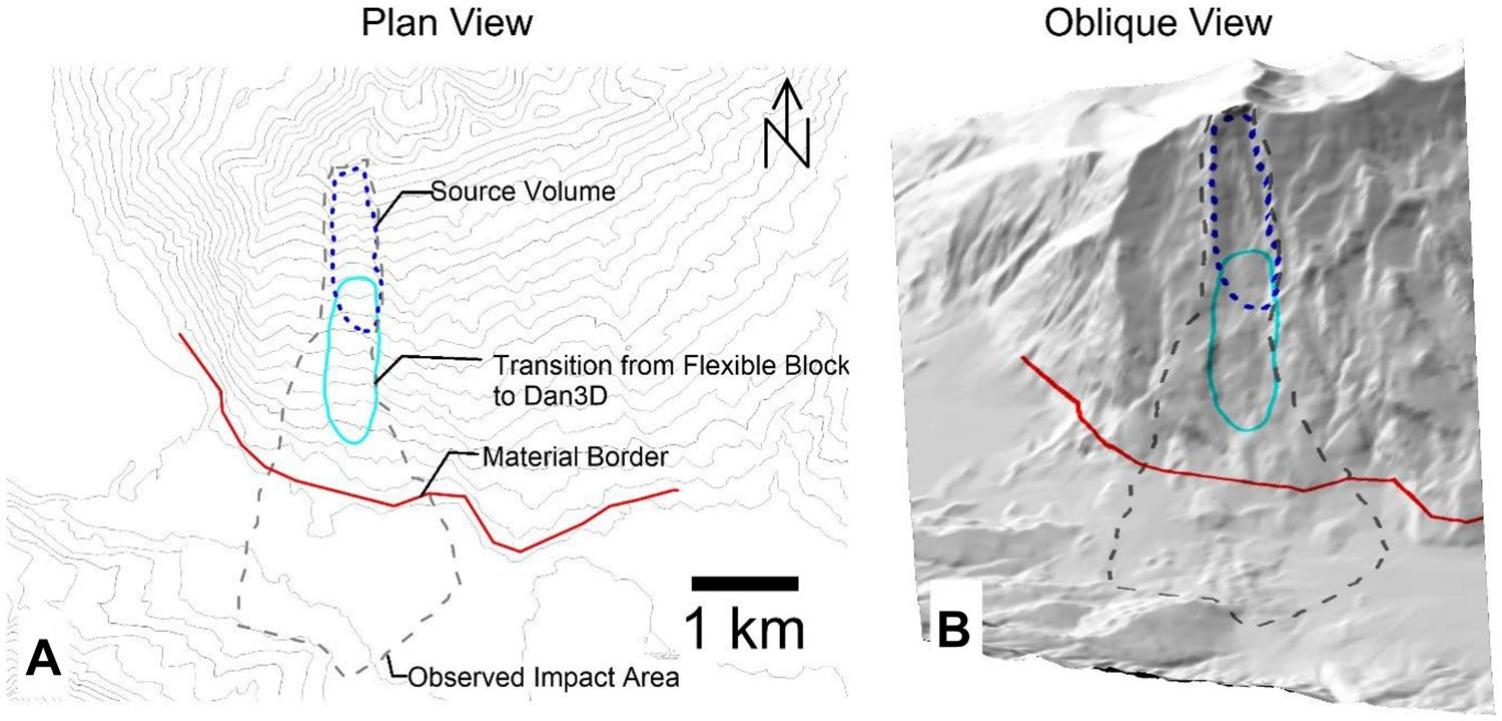
Example model set-up showing model parameterization. **A** plan view, **B** oblique view. A change of material occurs downslope of the red material border, as the rock avalanche overruns saturated sediments

When performing a back-analysis with Dan3D-Flex, a number of model inputs must be selected and/or calibrated. With reference to Fig. 3, these parameters are as follows:

1. The initial spatial extent and depth distribution of the failed mass. This is typically assessed through comparison of pre- and post-event digital elevation models, or a reconstruction of the pre-failure topography based on geomorphic evidence. Uncertainties in this input are not addressed in the present work, but can be considered based on the methods presented in Jaboyedoff et al. (2020).
2. The location and extent of any basal rheology changes (e.g., due to a change in the material type encountered along the path, as described in Fig. 1).
3. The values of parameters that govern all basal rheologies used, which will be denoted by the parameter vector ${\varvec{b}}$.
4. The rigid motion time, which is the amount of time that the simulation is governed by the flexible block model, before flow-like behavior occurs (Aaron and Hungr 2016b). In Fig. 3, the cyan outline shows where the mass is located when flow-like behavior is specified to occur for the example case shown.
5. The volume and spatial distribution of entrainable substrate. As described in McDougall and Hungr (2005), this can be estimated based on geomorphological mapping and is not described in the present work.

Previous work has shown that simulation results are insensitive to the user specified value of the internal friction angle, so long as it is kept within a reasonable range (Hungr 1995, 2008). Additionally, Aaron and Hungr (2016b) show that simulation results are insensitive to the rigid motion time, so long as the landslide mass has mostly vacated the rupture surface at the time it switches to fluid behaviour. Thus, of the five parameters specified above, ${\varvec{b}}$ exerts the strongest control on the numerical model results, and it is for this reason that model calibration is focused on constraining ${\varvec{b}}$.

The parameters typically calibrated in a back-analysis are those that govern the basal rheology and the associated basal shear resistance term, ${\tau }_{zx}$. These basal rheologies represent the bulk resistance to motion that arises between the flowing fragments of rock and the material they overrun, which varies depending on the location along the runout path (Fig. 1). Dan3D-Flex features an open rheological kernel, so that a variety of rheologies can be used to simulate different resistance behavior exhibited by the source and path material. An overview of these rheologies is provided in Hungr and McDougall (2009), and only the Voellmy and frictional rheologies will be briefly summarized here.

The Voellmy rheology is given by (e.g., Koerner 1976; Hungr and Evans 1996):

1 $${\tau }_{zx}=-{(\sigma }_{z} f+\frac{\rho g{v}_{x}^{2}}{\xi })$$

where ${\sigma }_{z}$ is the bed normal total stress, $\rho$ is the density, $g$ is gravitational constant, ${v}_{x}$ is the velocity in the direction of motion, *f* is the friction coefficient, and *ξ* is the turbulence coefficient. Both *f* and *ξ* are calibrated parameters, or fitting coefficients, in the equivalent fluid context, and are selected based on the probabilistic calibration procedure detailed below. The friction coefficient, *f*, is a bulk value that represents the apparent friction coefficient between the moving rock avalanche and the substrate it moves over. The velocity-dependent term in Eq. (1), whose magnitude is governed by *ξ,* commonly referred to as the turbulence coefficient, is used to account for all forms of velocity-dependent resistance, including potential pore fluid effects, which may be induced by rapid undrained loading (Hungr and Evans 2004)*.*

The frictional rheology, based on Coulomb frictional behavior, is given by:

2 $${\tau }_{zx}= {-\sigma }_{z} \mathrm{tan}({\varnothing }_{b})$$

where ${\varnothing }_{b}$ is the calibrated bulk friction angle, which includes pore-pressure effects.

For the back-analyses presented in this paper, we parameterized the source zone using the frictional rheology, sedimentary path material with the Voellmy or Bingham rheology (the Bingham rheology, which assumes the rock avalanche acts as a Bingham fluid, governed by a velocity dependent viscous resistance and constant yield stress, is described in Hungr and McDougall 2009) and bedrock with the frictional rheology. A distinction is made between source zone and path material resistance, because the simulated rock avalanche is moving over different materials, and is therefore likely experiencing different basal resistance (Aaron and McDougall 2019). This parameterization methodology is consistent with previously successful back-analysis using semi-empirical runout models (e.g., Geertsema et al. 2006; McDougall et al. 2006; Aaron and Hungr 2016a; Moore et al. 2017).

Calibration uncertainty, defined in the introduction, results from an inability to accurately resolve the rheological parameters (e.g., *f* and *ξ* in Eq. (1)) based on a given set of field measurements. When the frictional rheology is used, there is little calibration uncertainty in the results, as back-analyses result in a single, best-fit friction angle. However, when the two parameter Voellmy or Bingham rheologies are used, there can be non-uniqueness and parameter correlation in the calibrated results, which must be accounted for when making forecasts.

Therefore, for the $N=22$ case histories where we used the Voellmy or Bingham rheologies (selected based on the substrate material considerations detailed above), we determined a posterior distribution of the model parameters using the Bayesian inference techniques detailed in Aaron et al. (2019). This calibration methodology quantifies the quality of a simulation in terms of a fitness metric that can flexibly account for a variety of constraints, including impact area, velocity, and deposit distribution, depending on data availability.

For each case, the fitness has been computed for a wide range of model parameter combinations. Parameter combinations are selected by systematically sampling the parameter space at regular intervals over the entire plausible parameters space. The model misfit for each parameter combination were then transformed into the model parameter’s posterior density distribution, which quantifies calibration uncertainty and is denoted as ${{\uppi }^{\left(i\right)}}_{post}\left({\varvec{b}}|{\boldsymbol{ }{\varvec{r}}}^{({\varvec{i}})}\right)$. Here, ${\varvec{b}}$ is the vector of input parameters (for example, $f$ and $\xi$ in Eq. (1)) and ${\varvec{r}}$ is the vector of available observations with respect to which the misfit is quantified. The superscript $i$ indicates that the calibration is done on a case by case basis. Hence, we have $N$ distinct posterior distributions for $N$ cases in the database. A detailed discussion of the procedure to determine the posterior density distribution for a given case history is provided in Aaron et al. (2019).

More complex model parameterizations, which use more than two materials, could potentially be considered for both back-analysis and forward prediction. The use of more complex models very likely means a larger number of model parameters, which necessitate different calibration techniques, for example, machine-learning techniques such as Shahri et al. (2021). In this scenario, the grid search used to derive the parameter posterior distributions in this study would become too computationally intensive. Here, we minimize the number of input parameters, so that they can be constrained using field observations and our database of 31 case histories, as will be described in the "Assessment of mechanistic uncertainty" section.

### Calibration results and discussion

**Fig. 4:**
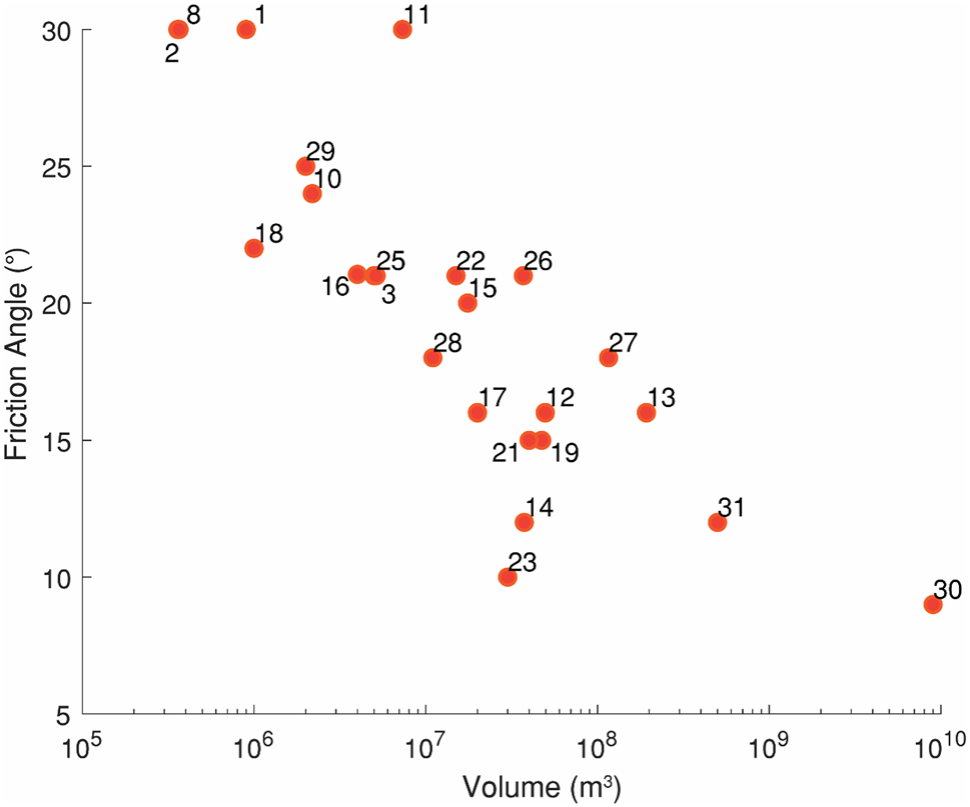
Back-analyzed friction angles in the source zone. See Fig. 2 for case names that correspond to the case numbers. Figure modified after Aaron and McDougall (2019), with two new cases added (30 and 31)

The probabilistic back-analysis method described above has been applied to all cases in our database. A summary of all the back-analyzed cases, including references to previous work, the back-analysis parameterization, available constraints, and path materials is presented in Supplementary Table S2. For cases where the frictional rheology was used in the source zone, the back-analyzed friction angles are shown in Fig. 4. As summarized in Aaron and McDougall (2019), these results show a volume dependent trend, which demonstrates a systematic reduction of basal resistance in the source zone as volume increases.

**Fig. 5:**
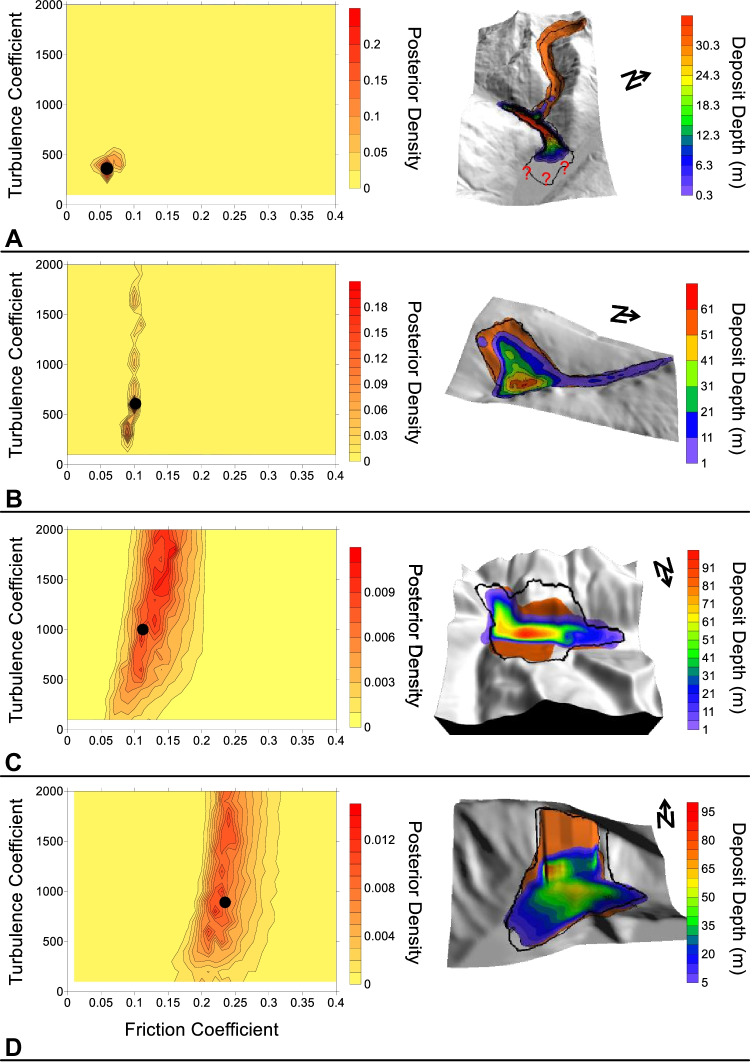
Example calibration results when the path material is sediment and parameterized using the Voellmy rheology. **A** Mt Meager, with a contour interval of 0.025. **B** McAuley Creek, with a contour interval of 0.01. **C** Avalanche Lake, with a contour interval of 0.001. **D** Madison Canyon, with a contour interval of 0.001. The black dot indicates the parameter set that results in simulations that best match the constraints. The units of the turbulence coefficient are m/s^2^. The black line on the best fit simulation results shows the observed impact area

Four example parameter posterior densities for cases where the path materials were parameterized with the Voellmy rheology are shown in Fig. 5. All other posterior distributions are presented in the Supplementary Information. The abscissa of the posterior distributions shown in Fig. 5 is the friction coefficient, and the ordinate is the turbulence coefficient, described in Eq. (1). The contoured value is a measure of the goodness of fit that results when the corresponding friction and turbulence parameters are inputted into Dan3D for the given case history.

For cases where bedrock was encountered as the path material, the back-analyzed friction angles are shown in Table 1. These angles are representative of the bulk basal resistance experienced by the simulated rock avalanche as it moved over the bedrock substrate. For one case, the West Salt Creek Rock Avalanche, the Bingham rheology was found as the best-fit rheology. As summarized in Aaron et al. (2017), this is thought to be because this case overran saturated fine grained material, whose shear behavior can be described by the Bingham rheology.

**Table 1:** Best-fit friction angles for cases that overran bedrock. The value for Thurweiser is based on Sosio et al. (2008), Chehalis is based on Si et al. (2018), Daubensee is based on Grämiger et al. (2016), and Bingham Canyon is modified based on Moore et al. (2017)

**Case name (case number)**	**Volume (M m**^**3**^**)**	**Path material friction angle (°)**
Thurweiser (10)	2.2	24
Chehalis (29)	2	25
Daubensee (25)	5.1	21
Bingham Canyon (23)	30	20

Our results show that there is minimal variance (and therefore minimal calibration uncertainty) in the back-analyzed source zone friction angles, as well as the best-fit friction angles for cases that overran bedrock as the path material. This is because the source zone friction angles are well resolved by the presence or absence of deposition in the source zone (e.g., Moore et al. 2017), and the observed impact area well resolves best-fit friction angles along the path (e.g., Grämiger et al. 2016). However, significant calibration uncertainty must be considered when making forecasts of potential rock avalanches that overrun sedimentary substrates, as shown in Fig. 5C, D.

## Assessment of mechanistic uncertainty

**Fig. 6:**
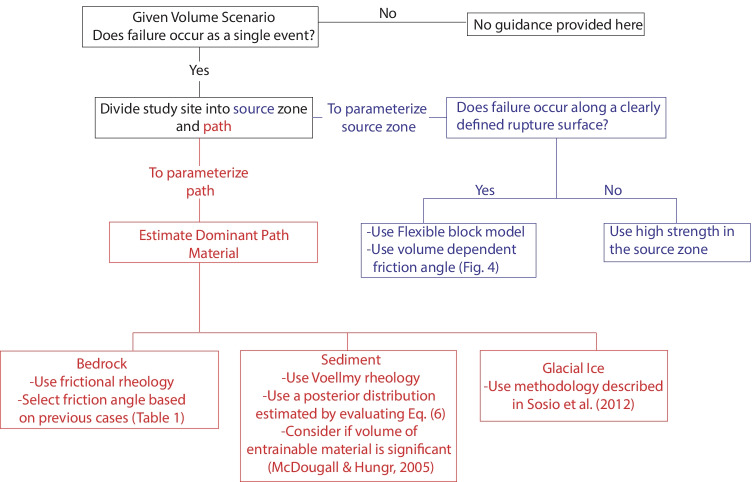
Decision tree to guide the selection of parameters for the prediction of rock avalanche runout using Dan3D-Flex. The blue branch indicates the decision process for parametrizing the source zone, and the red branch indicates that for the path

In this section, we provide a decision tree that can guide the assessment of the similarity of a potential future rock avalanche to the back-analyzed cases in the database, and therefore address mechanistic uncertainty associated with forecasting rock avalanche motion. The parameters required to make a forward prediction with a semi-empirical numerical runout model are the same as those required for back-analysis (summarized in Fig. 3); however, when making predictions the parameters can no longer be calibrated. Two of these inputs, the thickness and distribution of the initial failure, and the availability of entrainable sediment, must be estimated based on separate geomorphological, structural, and/or slope stability analyses (McDougall and Hungr 2005; Jaboyedoff et al. 2020) and will not be described in the present paper. All other parameters can be selected by assessing the similarity of the case of interest to the cases in the database, as described on the decision tree shown in Fig. 6.

The first node in Fig. 6 represents the possibility of catastrophic failure, defined as sudden failure of the entire unstable volume. Catastrophic failure is contrasted with piecemeal failure, where the unstable volume unravels. For cases that fail in a piecemeal manner, it is expected that runout will be much shorter than if catastrophic failure occurs. An example of piecemeal failure is the Randa rockfall, where 30 M m^3^ of rock unravelled in a series of small volume failures, creating a large talus cone at the base of the slope (Eberhardt et al. 2004). The decision framework presented in Glastonbury and Fell (2008) can be used to guide the assessment of the likelihood of catastrophic failure. Based on the available evidence, all of the cases analyzed in the present work failed catastrophically, so no guidance can be given about assessing the runout of a piecemeal failure mechanism on the basis of these cases.

If failure is catastrophic, the next step given in Fig. 6 is to separate the study site into the source zone and path (these zones are shown in Figs. 1 and 3, and described in the introduction). Guidance for how to make this division can be taken from the cases in the databases. For Huascaran, Thurweiser, Bingham Canyon, Nomash River, Madison Canyon, Mt Meager, Avalanche Lake, Guinsaugon, Rinderhorn, Flims, Rautispitz, and Platten, the location of the rheology change is easy to predict as there is a clear distinction between the source and path materials. Six des Eaux, Val Pola, Mystery Creek, Goldau, Molveno, and Frank all descended steep source slopes before spreading out on a valley floor. For these cases, good results were found by using the source zone resistance parameters until the rock avalanches encounter valley floor sediments. For Daubensee and Chehalis, the path material was bedrock, so no change in rheology was implemented between the source zone and path. The only case that did not fit well into this framework was McAuley Creek. As summarized in Aaron (2017), this could be due either to plowing of potentially entrainable sediments, or to multiple failures.

Following the subdivision of the study site into source and path, the relevant parameters for each of these material zones need to be specified. For the source zone (blue branch in Fig. 6), the parameters should be evaluated based on an analysis of the potential rupture surface. If failure is expected to occur along a continuous planar feature, then the flexible block model should be used. As summarized in Aaron and Hungr (2016b), the rigid motion distance can be selected based on an examination of the pre-failure topography. Aaron and Hungr (2016b) also showed that the final predicted impact area is insensitive to the choice of rigid motion distance, and good results have been found by specifying that fragmentation occurs when most of the failed mass has vacated the source zone (e.g., Aaron and Hungr 2016a; Aaron et al., 2017; Castleton et al. 2016; Grämiger et al. 2016; Moore et al. 2017). Therefore, the rigid motion distance can be selected a priori and it is not recommended that this parameter be varied in a probabilistic context. If failure is along an irregular surface then the flexible block model should not be used, as fragmentation is likely to occur close to the onset of failure.

Based on the calibration results, the frictional rheology should be used in the source zone, and the friction angle can be assigned based on the failure volume, which must be estimated prior to performing a runout analysis. Mechanistic uncertainty is limited when selecting this parameter, which can be done based on a lower bound or linear fit to the values in Fig. 4, depending on how much conservatism is desired for a particular forecast. For smaller volume cases, the back-analyzed frictional resistance corresponds to a friction angle a few degrees less than the limit equilibrium value, determined based on the dip of the rupture surface. This corresponds to the strength required for a catastrophic failure to initiate.

The red path in Fig. 6 is used to parameterize the path materials. If the path material is glacial ice, then parameters can be selected based on Sosio et al. (2012). For bedrock, the frictional rheology should be used to parameterize basal resistance, and preliminary guidance for the value of the friction angle can be taken from Table 1. For probabilistic predictions, a range of friction angles can be used; however, due to the limited number of case histories in the database that overran bedrock, it is presently difficult to give guidance on the probabilities to assign to these values, so some subjective judgment is needed.

For cases that overrun sediments, the selection of resistance parameters along the path is a highly uncertain part of a forward runout prediction, and expert judgement is presently required to assess the similarity of a potential future rock avalanche to a calibrated case history in the database. Therefore, we propose a statistical methodology to combine the results of back-analyses into a probability density function, which can then be used to make probabilistic estimates of rock avalanche runout. This methodology is summarized in the following section.

## Probabilistic prediction framework

In this section, we detail a predictive simulation framework that accounts for both data induced and mechanistic uncertainties, and can be used to parameterize the path material. Similar methodologies have been applied for other processes, such as snow avalanches and landslide generated waves (e.g., Straub and Grêt-Regamey 2006; Mergili et al. 2018a; Fischer et al. 2020). This section focuses on cases that overrun sediments, as they represent the majority of cases in the database; however, the same methodology can be applied to other types of substrate if the appropriate calibration data becomes available.

In this framework, we propose an expert-based prediction method that aims at leveraging relevant information from the complete event database. We will denote the outcome or event of interest for a future rock avalanche as ${\varvec{E}}$, which could be related to any attribute that must be predicted to assess rock avalanche risk, for example, that the future rock avalanche has an impact at a certain location or that the runout exceeds that particular location. It is also possible to extend the methodology to estimate the predictive distribution of other quantities of interest such as the overall run-out area and/or the velocity at a certain location. However, in the present work, we will only assess impact probabilities and the resulting runout exceedance probabilities, as these are often the most important parameters to obtain from a runout model in a rock avalanche risk analysis.

The predictive probability for the event ***E***, given the observations available for the cases in the database, is denoted by ${p}_{pred}\left({\varvec{E}}|{{\varvec{r}}}^{(1)},\boldsymbol{ }\dots ,\boldsymbol{ }{{\varvec{r}}}^{\left(N\right)}\right)$ and can be determined by:

3 $${p}_{pred}\left({\varvec{E}}|{{\varvec{r}}}^{(1)},\boldsymbol{ }\dots ,\boldsymbol{ }{{\varvec{r}}}^{\left(N\right)}\right)= \int p\left({\varvec{E}}|\boldsymbol{ }{\varvec{b}}\boldsymbol{ }\right){\uppi }_{post}\left({\varvec{b}}\boldsymbol{ }|\boldsymbol{ }{{\varvec{r}}}^{(1)},\boldsymbol{ }\dots ,\boldsymbol{ }{{\varvec{r}}}^{\left(N\right)}\right)\boldsymbol{ }d{\varvec{b}}$$

The first term in the integral, $p\left({\varvec{E}}|\boldsymbol{ }{\varvec{b}}\right)$, represents the probability of a future outcome given the parameter vector, and can be evaluated by examining numerical model outputs when parameter vector ${\varvec{b}}$ is used as input. As Dan3D-Flex is not a probabilistic model, when it is run for a particular parameter vector the output is deterministic (for example, an impact at a certain location will or will not be simulated to occur). However, there is some uncertainty in this deterministic outcome, as Dan3D-Flex will not perfectly reproduce an observed impact area when the optimal parameter set is chosen (this can be seen by comparing measured and modelled impact areas in Fig. 5). In the present work, we do not explicitly consider this source of uncertainty, and assign a value of $p\left({\varvec{E}}|\boldsymbol{ }{\varvec{b}}\right)$ = 1 to all areas where impacts are simulated to occur, and a value of $p\left({\varvec{E}}|\boldsymbol{ }{\varvec{b}}\boldsymbol{ }\right)=0$ to all locations outside of the simulated impact area. Further examination of the $p\left({\varvec{E}}|\boldsymbol{ }{\varvec{b}}\right)$ term is provided in the discussion.

The second term in Eq. (3), ${\uppi }_{post}\left({\varvec{b}}\boldsymbol{ }|\boldsymbol{ }{{\varvec{r}}}^{(1)},\boldsymbol{ }\dots ,\boldsymbol{ }{{\varvec{r}}}^{\left(N\right)}\right)$, is the parameter’s posterior density based on evidence given by the combined set of available data of all events in the database. In an idealized situation, in which there is reason to believe that all cases in the database follow the same underlying parameter distribution, and additionally that cases are independent and observations without error, the combined posterior is simply given by a normalized product of the individual posteriors. In reality, however, we are facing large levels of mechanistic uncertainties and sparse, potentially erroneous observations, which make this type of Bayesian inference unfeasible for our situation. More specifically, when comparing the back-analyzed posterior parameter distributions for the cases in the database (see Supplementary Information), two posterior distributions seldom coincide. This is likely caused by differences in the mechanism(s) governing movement of the individual cases.

Following this observation, we hypothesize that the case histories in the database sample various rock avalanche movement mechanisms that occur along the path, and that potential future events will behave similarly to a subset of these cases. A few examples of potential mechanisms include effects from confinement (Strom et al. 2019; Mitchell et al. 2020b), liquefaction of path material (Buss and Heim 1881; Hungr and Evans 2004), fragmentation of the rock avalanche material (Davies et al. 1999), and/or plowing of entrainable substrate (McDougall and Hungr 2005).

We formalize this hypothesis by partitioning all cases of the database into $N$ disjoint “classes,” referred to as ${{\varvec{c}}}^{(i)}$ for i = 1,…,N. Each “class” denotes the specific movement mechanism(s) that occurred along the path for a given case history, which combine to result in the case-specific posterior distribution. We furthermore denote the probability that the case we want to predict falls into class ${{\varvec{c}}}^{(i)}$ as $p({{\varvec{c}}}^{(i)})$. As the classes are disjoint, we have $\sum_{i=1}^{N}p\left({{\varvec{c}}}^{\left(i\right)}\right)=1$ and can use the law of total probability to break down the combined parameter’s posterior into:

4 $${\uppi }_{post}\left({\varvec{b}}\right|\boldsymbol{ }{{\varvec{r}}}^{(1)},\boldsymbol{ }\dots ,\boldsymbol{ }{{\varvec{r}}}^{\left(N\right)})=\sum_{i=1}^{N}{{\uppi }^{\left(i\right)}}_{post}\left({\varvec{b}}\right|\boldsymbol{ }{{\varvec{r}}}^{\left(i\right)})\boldsymbol{ }p({{\varvec{c}}}^{(i)})\boldsymbol{ }$$

in which ${{\uppi }^{\left(i\right)}}_{post}\left({\varvec{b}}\right|\boldsymbol{ }{{\varvec{r}}}^{\left(i\right)})$ is the model parameter posterior distribution for class ${{\varvec{c}}}^{(i)}$. The formula in Eq. (4) can be understood as a weighted average of the back-analyzed posterior distributions with weights given by the probabilities that a future event falls into one of the N classes. At present, we only consider evaluating Eq. (4) for the case where the path material is sediment, using the parameter posterior distributions given in the Supplementary Information. This procedure is equally applicable to cases that overrun bedrock and glacial ice; however, the best fit parameters for these case histories are generally given by a single, best-fit friction angle (e.g., Sosio et al. 2012; Aaron 2017). Therefore, ${{\uppi }^{\left(i\right)}}_{post}\left({\varvec{b}}\right|\boldsymbol{ }{{\varvec{r}}}^{\left(i\right)})$ in Eq. (4) would be given by a single, best fit friction angle.

In order to assess the probability that a potential future rock avalanche falls into a given class, we will explore an approach based on expert judgement, that can take guidance from past experience with landslide run-out modeling (Sassa 1985; Hungr et al. 2002; Hungr and Evans 2004; Aaron and McDougall 2019). Experts can assign the probability that the predicted event falls into class $p\left({{\varvec{c}}}^{\left(i\right)}\right)$. A simple way of doing this is to subjectively determine the similarity between events in the database and the case that is to be predicted and to denote it ${w}_{i}$. For an arbitrary similarity scale, the desired probability is then given by:

5 $$p\left({{\varvec{c}}}^{\left(i\right)}\right)=\frac{{w}_{i}}{\sum_{j=1}^{N}{w}_{j}}$$

Substituting this into the previous analysis yields the combined posterior probability distribution:

6 $${\uppi }_{post}\left({\varvec{b}}\right|\boldsymbol{ }{{\varvec{r}}}^{(1)},\boldsymbol{ }\dots ,\boldsymbol{ }{{\varvec{r}}}^{\left(N\right)})= \frac{\sum_{i=1}^{N}{{\uppi }^{\left(i\right)}}_{post}\left({\varvec{b}}\right|\boldsymbol{ }{{\varvec{r}}}^{\left(i\right)})\boldsymbol{ }{w}_{i}\boldsymbol{ }}{\sum_{i=1}^{N}{w}_{i}}$$

**Fig. 7:**
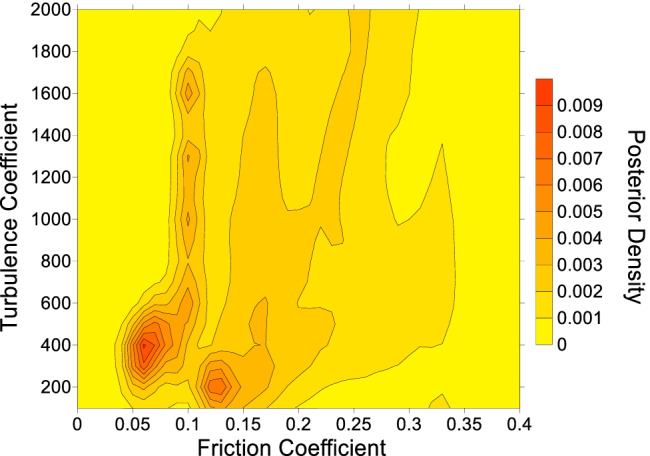
Probability density function derived from combining the best-fit Voellmy parameters from all the case histories. A description of the posterior probability density is provided in the "Probabilistic calibration methodology" section, as well as Aaron et al. (2019). The contour interval is 0.001

Note that Eq. (6) naturally reduces to the arithmetic average if a similarity value ${w}_{i}=1$ is chosen for each case in the database. The result of evaluating this arithmetic average is shown in Fig. 7. In Fig. 7, the high probability zones correspond to places where the best-fit parameters of many case histories overlap (this can be seen by comparing Fig. 7 to the posterior distributions given in the Supplementary Information). As discussed previously, the presence of multiple high probability zones in Fig. 7 is likely caused by different movement mechanism(s) occurring along the path.

## Evaluation of forecasting methodology

We have evaluated our methodology by applying it to the Frank Slide and the Turnoff Creek rock avalanche (Cruden and Krahn 1978; Beguería et al. 2009), using the methodology detailed below. We selected these cases because they have different confinement conditions and volumes, and therefore represent a sampling of rock avalanche types that may be encountered when performing rock avalanche risk assessment.

When applying our methodology to these two case histories, we excluded any case-specific information about the basal resistance parameters prior to deriving source zone friction angles and posterior probability density functions for the analyses (i.e., the cases were excluded from the calibration data for a cross-validation). However, we did not vary the source volume, the location of the boundary between source and path material, or the rigid motion distance. Further, as these two cases have already occurred, we are able to compare the observed runout to the simulated results. We therefore term the resulting probabilistic results as “pseudo-forecasts,” both because we are simulating events that have already occurred, and because we do not explore all possible sources of uncertainty. Further validation of this method, exploring more sources of uncertainty, is the subject of ongoing work.

For each of the pseudo-forecasts, we used four different posterior parameter distributions for the path materials, calculated using Eq. (6), to derive probabilistic forecasts of impact area. The use of four different distributions enabled testing of the sensitivity of forecast results to the selected subjective weightings, as well as the influence of both calibration and mechanistic uncertainty on forecasting results.

The four different posterior distributions used in Dan3D-Flex are described in Table 2. Scenario POS (case posterior) in Table 2 was selected to isolate the influence of calibration uncertainty on forecasting results, as the use of the case specific posterior only accounts for uncertainties in the calibration data. The next three scenarios explore the sensitivity of forecasting results to increasing mechanistic uncertainties, with EXP (expert judgement) having less mechanistic uncertainty than EQL (equally weighted posteriors), and EQL less than UNI (uniform distribution over parameter space). The UNI scenario represents the scenario of making forecasts in the absence of a database of calibrated parameters for the path material.

**Table 2:** Descriptions of Dan3D-Flex numerical scenarios and PRE-RA empirical scenarios used to test the forecasting methodology

**Scenario name**		**Description**
Dan3D-Flex Numerical Model Scenarios	POS	Posterior distribution for the case determined from its own back-analysis
	EXP	Expert judgement to weight the case histories in the database
	EQL	Equal weighting of all case histories
	UNI	Uniform distribution for the parameter space (database of calibrated case histories not taken into account)
PRE-RA Empirical Model Scenarios	No Confinement Variables	Regression only based on volume and fall height
	C = 0	Regression based on volume, fall height and unconfined topography
	C = 1	Regression based on volume, fall height and confined topography

Probabilistic simulation results were obtained by first pre-computing the model results for a regular grid of parameter combinations. For the selected case histories, values of the friction coefficient were selected between *f* = 0.05 to 0.30 with steps of 0.01 and turbulence coefficients were selected between $\xi$ = 100 m/s^2^ and 2000 m/s^2^ with steps of 100 m/s^2^. This range of parameters was selected as it encompasses the most likely values of the parameters (Fig. 7), while keeping the number of simulations reasonable. The input posterior probability density functions were then randomly sampled 10^5^ times, and the pre-computed results corresponding to the randomly sampled parameters were combined to determine exceedance probabilities (defined as the probability of an event going a specified distance or further). A value of 10^5^ random samples was selected based on preliminary testing, which revealed that results stabilized using this value.

Forecasting results were further cross checked using “The Probabilistic Runout Estimator – Rock Avalanche (PRE-RA),” a statistical tool presented in Mitchell et al. (2020b). PRE-RA can be used to make probabilistic predictions of parameters such as runout length. This is achieved through the use of survival functions derived from linear regression using a database of 49 Canadian rock avalanche case histories. In the present work, the outputs of this model were compared to predictions made using Dan3D-Flex. Two regression equations were used to compare empirical-statistical and numerical predictions, which are described in Table 2. These included 1) a prediction of runout length based on volume and fall height, and 2) a prediction of runout length based on volume, fall height, and topographic confinement. These equations, and associated regression coefficients, were presented in Mitchell et al. (2020b).

### Frank slide

**Fig. 8:**
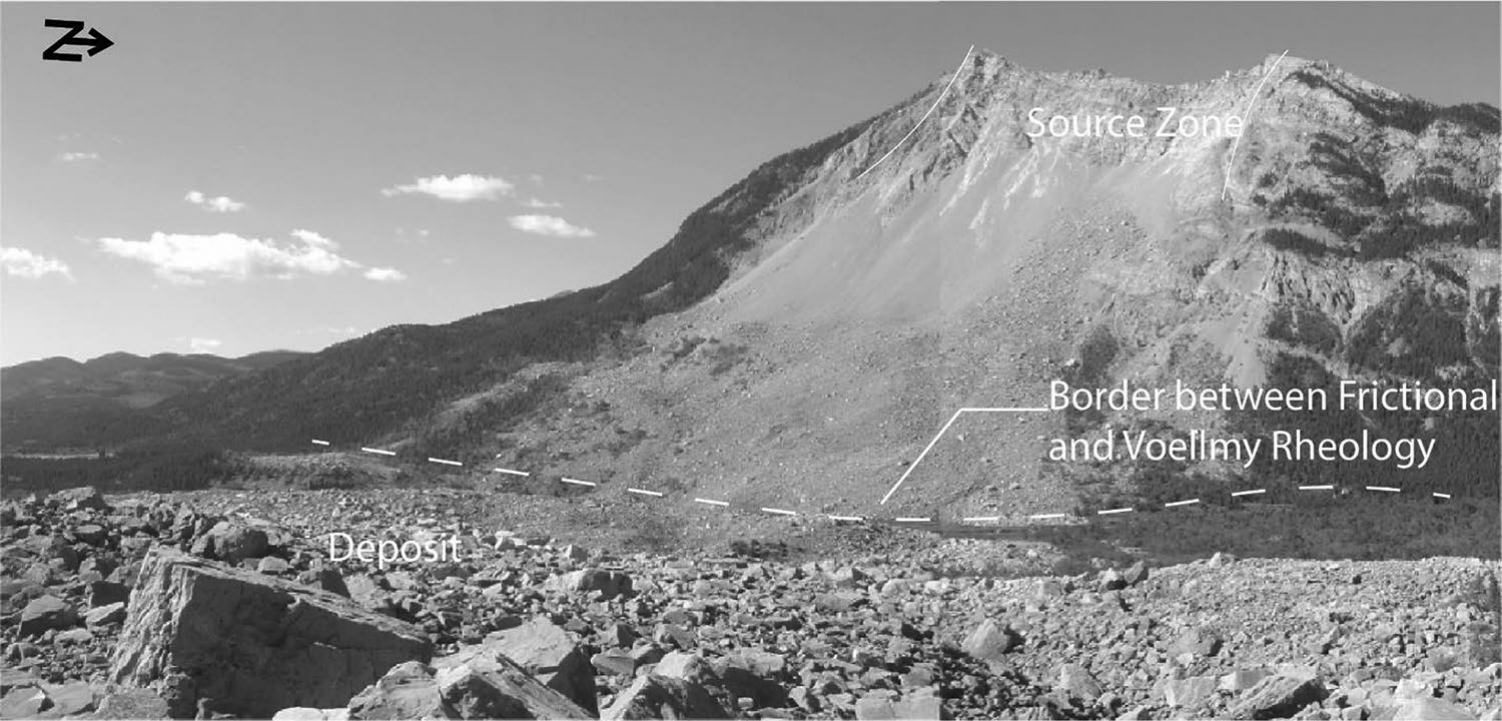
Overview of the Frank slide, showing the source zone, deposit, and material boundary used for the pseudo-forecast. Figure is modified after Aaron et al. (2019)

The Frank Slide was a rock avalanche that occurred in 1903 in Alberta, Canada. A number of previous studies have analyzed this event (Cruden and Krahn 1978; Cruden and Hungr 1986; McDougall 2006), so only a few details relevant to the present analysis will be presented. The topographic rasters used for the surface the rock avalanche travelled over, as well as the thickness of material in the source zone, are the same as that used in McDougall and Hungr (2004). Following the methodology summarized in Fig. 6, the path was separated into source and path zones, as shown in Fig. 8.

**Fig. 9:**
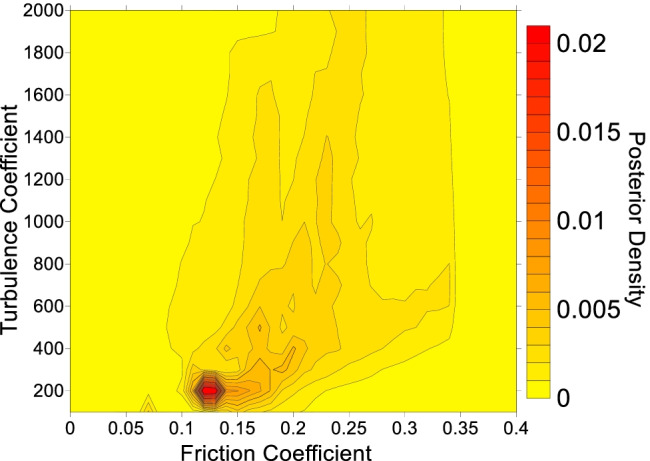
Posterior probability density function for the path material, used for the Frank Slide EXP scenario. The contour interval is 0.001

This event involved an estimated volume of 37 M m^3^, so a friction angle of 15° was selected for the source zone (Fig. 4). To construct the posterior probability distribution for the EXP scenario (Table 2), all cases were assigned equal weight, except for events that transitioned into debris avalanches/ debris flows (Huascaran, Nomash, Mt. Meager, Zymoetz and McAuley). Since the Frank Slide moved over unconfined topography, these cases were assigned a weight of zero. The resulting posterior PDF is shown in Fig. 9.

**Fig. 10:**
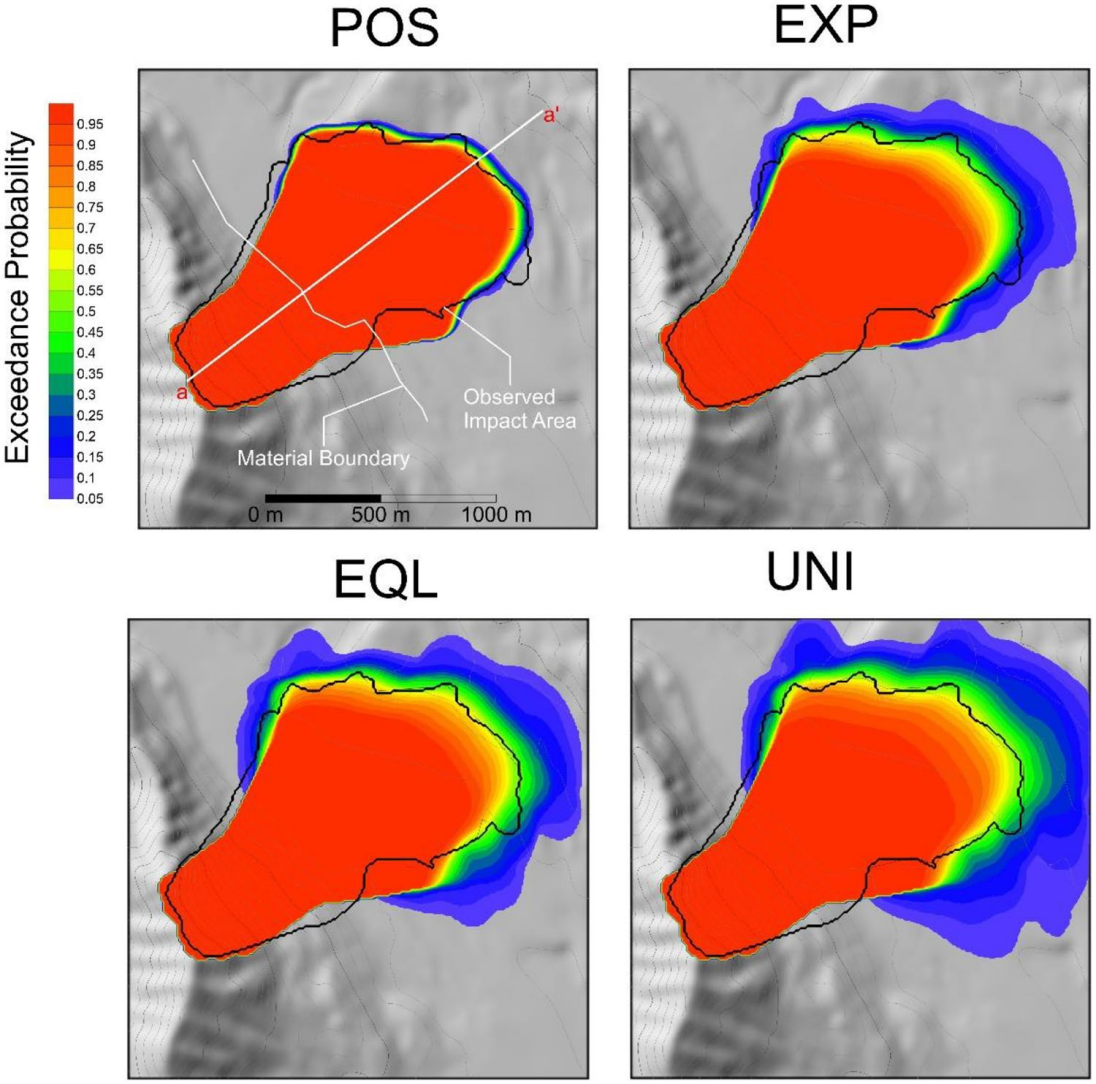
Probabilistic analysis of impact area for the Frank Slide using four different posterior probability distributions for the path material. POS) posterior distribution, EXP) expert judgment, EQL) equal weighting, UNI) uniform distribution. Refer to Table 2 for more detailed descriptions of the four scenarios

**Fig. 11:**
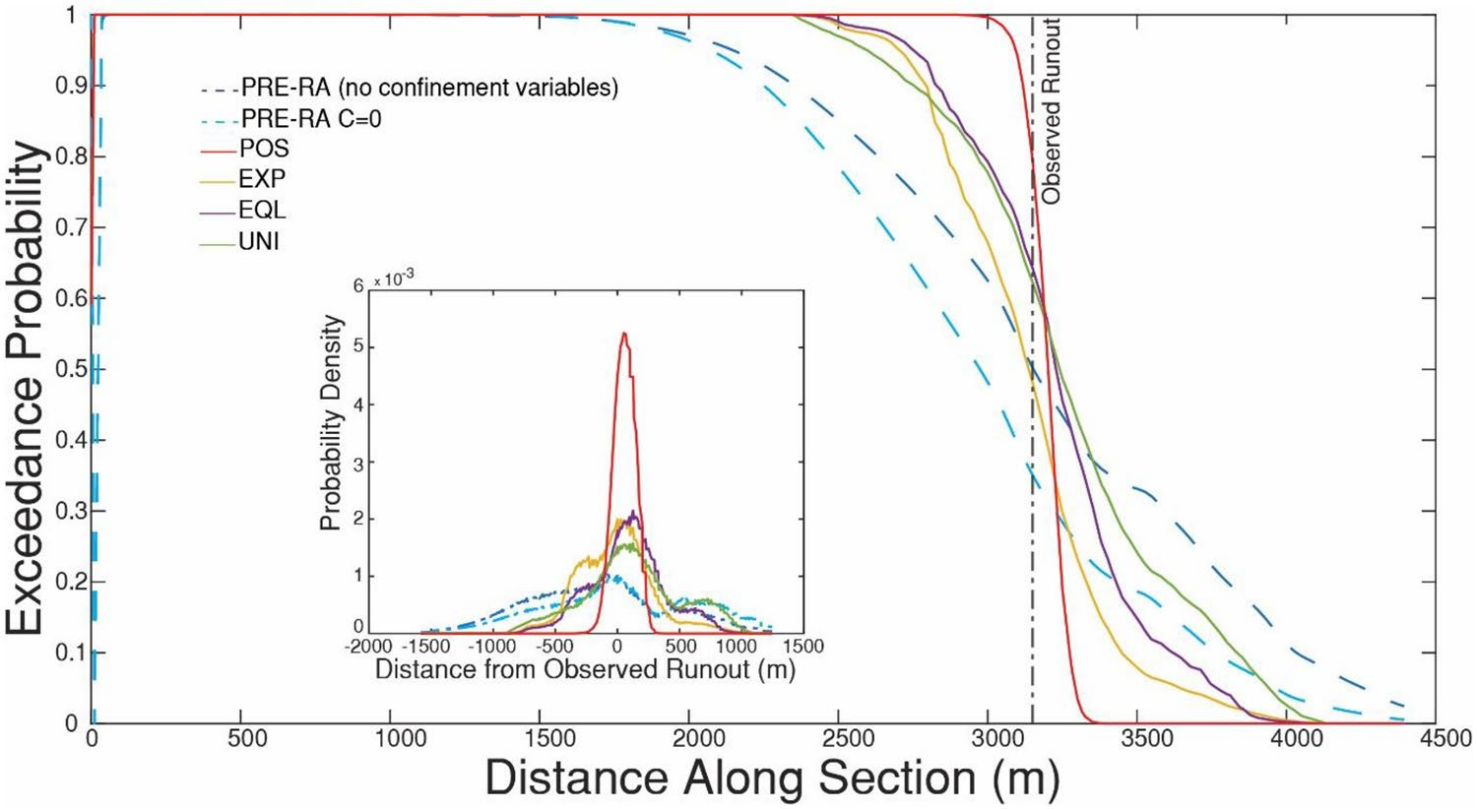
Comparison of numerical and empirical predictions for the Frank Slide. The section line used is labelled a-a’ in Fig. 10. Refer to Table 2 for descriptions of the four Dan3D-Flex numerical scenarios and two PRE-RA empirical scenarios. Further details on PRE-RA can be found in Mitchell et al. (2020b). The inset shows the probability density for predicted distance minus observed runout distance for the six methods, with zero representing a prediction that matches the observed runout distance, positive numbers an overprediction, and negative numbers an underprediction

The results of the probabilistic analysis of impact area are shown in Figs. 10 and 11. As can be seen in Fig. 10, all four methods of assigning weights for the parameter posterior distribution result in a significant probability of exceedance being assigned to the observed runout. The main difference between these methods is in the precision of the forecasts, with POS resulting in the most precise forecasts, followed by EXP, EQL, and UNI.

A similar result is seen in Fig. 11 when comparing the Dan3D-Flex and PRE-RA results. All methods assign a significant exceedance probability to the observed runout, with POS assigning the highest and the two PRE-RA methods the lowest. The inset in Fig. 11 shows the probability density of the predicted minus observed runout, which can be used to assess the precision of the pseudo-forecasts. PRE-RA predicts the largest spread about the observed runout (indicating that these forecasts are the least precise), followed by the Dan3D-Flex results.

### Turnoff creek

**Fig. 12:**
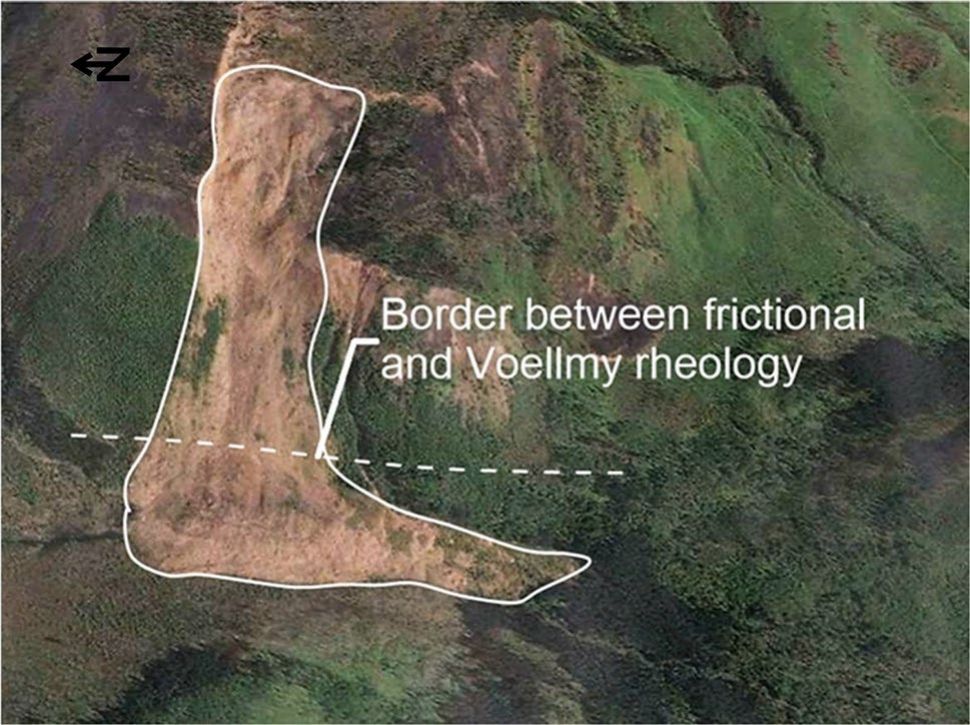
Overview of Turnoff Creek. Image: Google Earth, Digital Globe

The Turnoff Creek rock avalanche occurred in 1992 in British Columbia, Canada, and involved an estimated 4 M m^3^. It failed along a continuous bedding plane (Beguería et al. 2009). An overview of this rock avalanche is shown in Fig. 12. Following the methodology presented in Fig. 6, the study site was divided into source zone and path material, as shown in Fig. 12. Using the calibration data presented in Fig. 4, a friction angle for a 4 M m^3^ failure is expected to be approximately 21°. This value is a few degrees below the limit equilibrium angle and was selected in the source zone. A rigid motion distance was selected to correspond with the landslide fragmenting when most of the failed material had vacated the source zone.

**Fig. 13:**
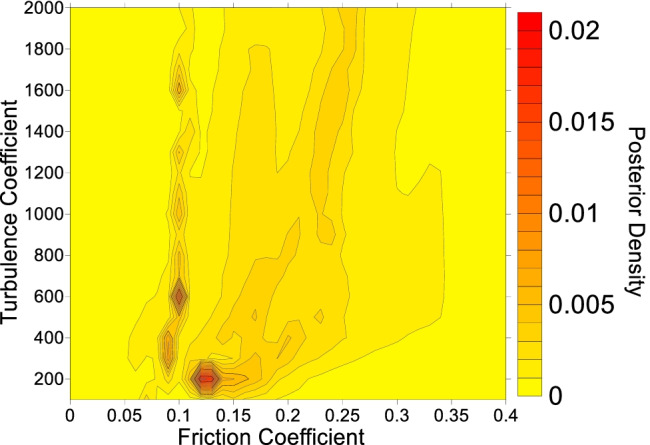
Posterior probability distribution for the Turnoff Creek rock avalanche path material parameters derived based on expert weighting of the cases in the database. The contour interval used is 0.001

After assigning a friction angle to the source area, the likely path materials were assessed. Beguería et al. (2009) reported that there was limited evidence of entrainment in the deposit, and the site was classified by Mitchell et al. (2020b) as having unsaturated sediment based on literature review and geomorphic interpretation. Based on this information, as well as the decision tree presented in Fig. 6, the path materials were parameterized using the Voellmy rheology. For the EXP scenario, all cases were equally weighted except for those that transitioned into a debris flow/debris avalanche. These cases were assigned lower, non-zero weights, based on the considerations of the path material detailed above. The resulting posterior PDF is shown in Fig. 13.

**Fig. 14:**
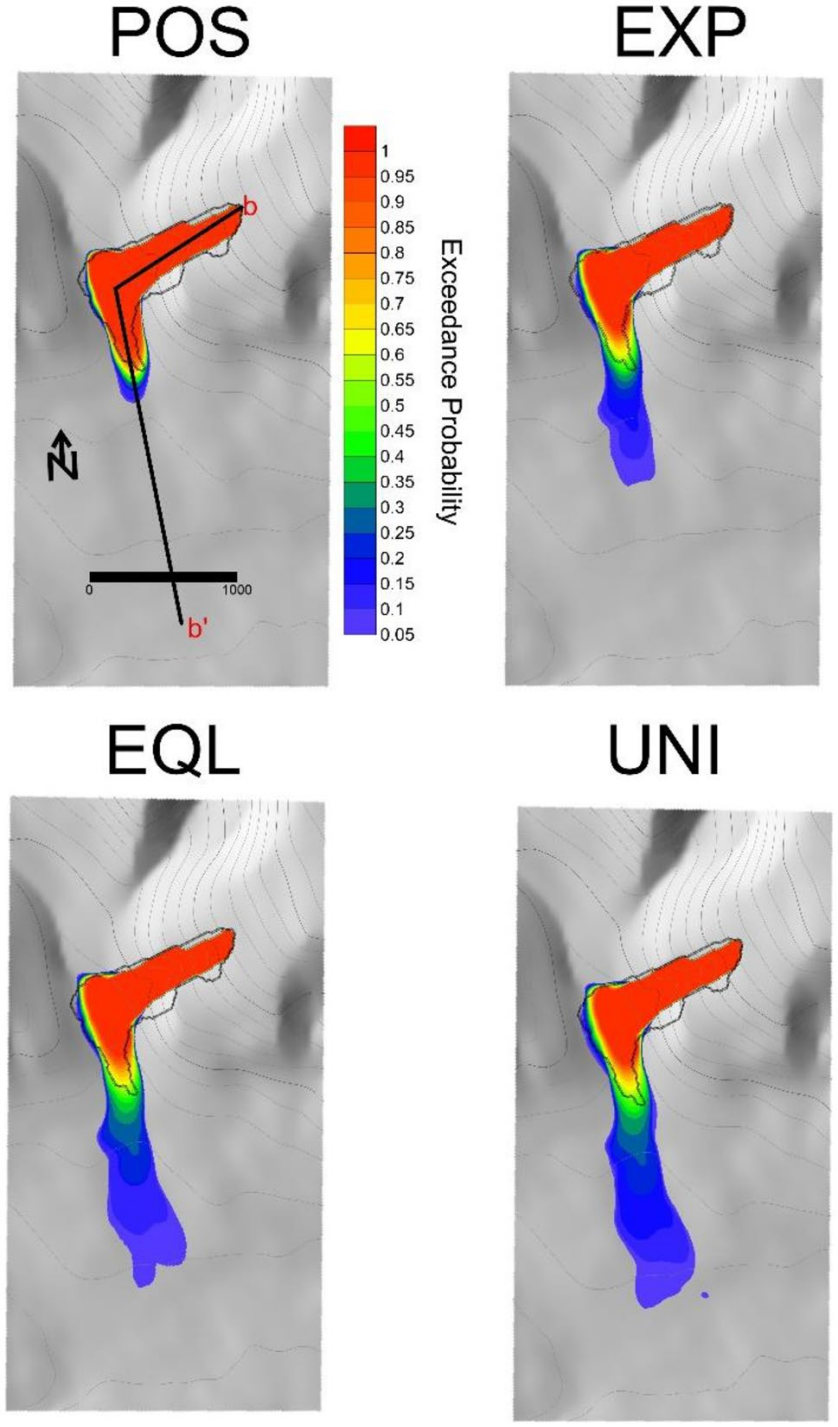
Probabilistic analysis of impact area for the Turnoff Creek rock avalanche using four different posterior probability distributions for the path material. POS) posterior distribution, EXP) expert judgment, EQL) equal weighting, UNI) uniform distribution. Refer to Table 2 for more detailed descriptions of the four scenarios

**Fig. 15:**
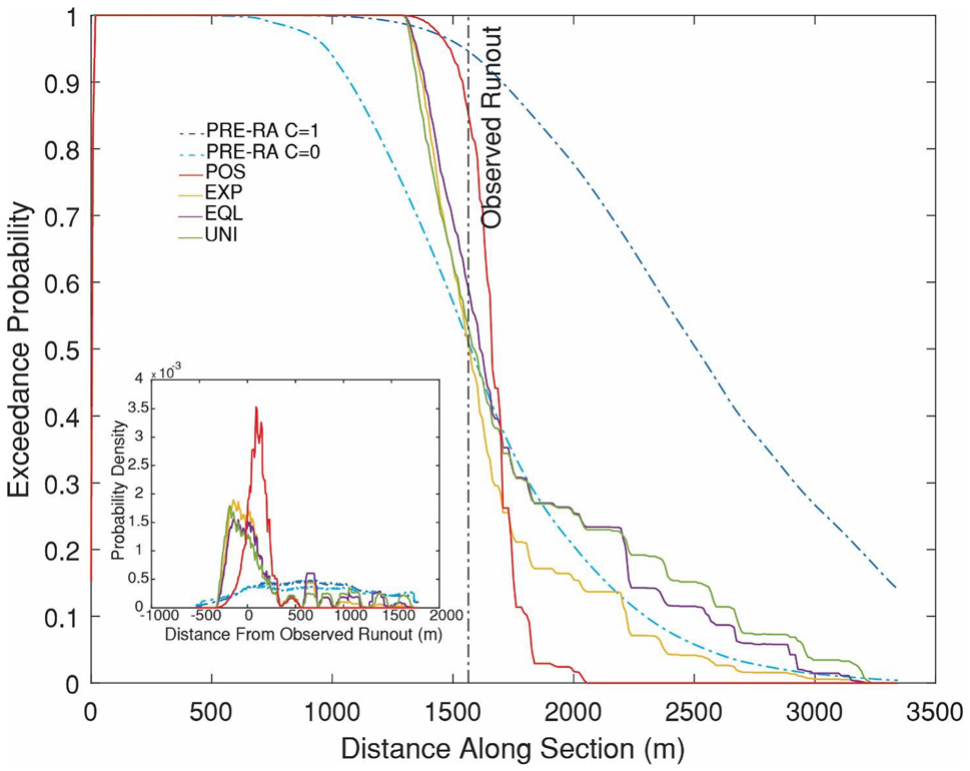
Comparison of numerical and empirical predictions for the Turnoff Creek rock avalanche. The section line used is labelled b-b’ in Fig. 14. Refer to Table 2 for descriptions of the four Dan3D-Flex numerical scenarios and two PRE-RA empirical scenarios. The inset shows the probability density for predicted distance minus observed runout distance for the six methods, with zero representing a prediction that matches the observed runout distance, positive numbers an overprediction, and negative numbers an underprediction

The resulting runout exceedance probabilities are shown in Figs. 14 and 15. Similar to the Frank Slide case, the POS simulations result in the most accurate and precise predictions, followed by the EXP, EQL, and UNI. The Fig. 15 inset shows that the PRE-RA results are less precise than the Dan3D-Flex results, but do assign a significant exceedance probability to the observed runout. Additionally, the PRE-RA predictions that include lateral confinement are much more conservative than all other forecasts. Prior to this case occurring, assessment of whether this case would have frontal or lateral confinement would likely be highly uncertain, so these predictions would have been difficult to definitively rule out.

## Discussion

The analysis framework presented here provides a method to make probabilistic forecasts of rock avalanche motion that fits naturally into the landslide risk analysis framework (e.g., Hungr 2016). This method accounts for both calibration and mechanistic uncertainty, which were quantified based on a database of 31 case histories. Calibration uncertainty was assessed by applying a probabilistic calibration methodology to each case in the database, and the results show that significant calibration uncertainty is only present when the path material is sediment. Additionally, minimal mechanistic uncertainty was found to be associated with the source zone basal resistance parameters, as they are well explained by volume. In contrast, significant mechanistic uncertainty exists when the path material is sediment. We thus propose a method, based on expert judgement, to account for calibration and mechanistic uncertainty in the path material parameters. We then investigated the accuracy, precision, and relative contributions of these two types of uncertainty by making pseudo-forecasts of two case histories that have already occurred.

Overall, we found that mechanistic uncertainty is greater than calibration uncertainty when predicting rock avalanche motion. For the two cases presented here, the range of potential runout was increased 4 to 5 times when mechanistic uncertainty is accounted for when making predictions (this can be seen by comparing the POS to EXP cases in Figs. 11 and 15). This highlights the need for further research into the mechanisms that control rock avalanche movement along the path. This would better constrain the similarity of future cases to those present in the database, reducing mechanistic uncertainty and resulting in more precise predictions. Additionally, while the cases in our database appear to be somewhat biased towards high mobility (Fig. 2), a future event may be more mobile than all of the cases in the database.

A key aspect of the proposed methodology is the use of expert judgement to assess how similar cases in the database are to a future case of interest. In practice, this will be difficult to do, especially when making a decision to exclude the more mobile cases in the database (due to the fact that the resulting predictions will be less conservative than if these cases are included). Figures 10 and 12 show that the results are relatively insensitive to this choice of weighting, so long as the uniform weighting is not used (which results in the most conservative predictions). Reasonably conservative forecasts can be made by equally weighting all case histories. These forecasts could then be refined by adding in the subjective weights, at the expense of some transparency in the forecast procedure.

Some guidance regarding the subjective weighting terms can be taken from the database of calibrated case histories. The cases with the lowest best-fit friction coefficients for the path material are Mt. Meager, Nomash River and Huascaran (Plafker and Ericksen 1978; McDougall and Hungr 2005; Guthrie et al. 2012). The debris involved in the Mt. Meager and Nomash River cases became channelized, and in both cases, the moving mass entrained significant quantities of surface water and loose saturated sediments and transformed into debris avalanches (McDougall and Hungr 2005; Guthrie et al. 2012). For forward analysis where the rock avalanche will not become channelized, these case histories can be assigned a low weight when deriving a posterior density function for the path material parameters. Systematic mobility enhancement due to channelization was also noted by Mitchell et al. (2020b). Similarly, the Huascaran rock avalanche became channelized, but also had its mobility enhanced by glacial ice in the source zone (Plafker and Ericksen 1978; Evans et al. 2009). It can therefore be assigned a low weight for forward analyses of cases that do not have these characteristics. Additionally, modern machine learning algorithms, which have been successfully applied to fields such as landslide susceptibility mapping (e.g., Zhang et al. 2019; Abbaszadeh Shahri and Maghsoudi Moud 2021; Zhou et al. 2021), could also be applied to assist with the selection of similarity parameters (Vasu et al. 2018). However, the paucity of available 3D input data in our current database limits the amount of data available to train these models. As the database grows, these methods may become more applicable in the future.

The two example pseudo-predictions were compared to those made using PRE-RA, an empirical-statistical tool. As shown in Figs. 11 and 15, PRE-RA and Dan3D-Flex provide complementary analyses of potential runout distance. Compared to PRE-RA, Dan3D-Flex tends to predict higher exceedance probabilities in the proximal path, which could potentially lead to extending of hazard zones associated with low return periods if a screening level analysis is further refined through numerical modelling. This difference is primarily caused by the use of a single friction angle in the source zone, selected based in Fig. 4, which governs the minimum runout distance predicted by the numerical model. As can be seen in Fig. 4, this parameter appears to be well correlated with volume and is thus not subject to significant mechanistic uncertainty.

Further differences between PRE-RA and the Dan3D-Flex results were noted for the Turnoff Creek case history. Figure 15 shows that assumptions made for confinement, which can be difficult to predict before an event, can have a strong influence on the runout lengths predicted by PRE-RA. The use of Dan3D-Flex, which explicitly considers path topography, can help to address this source of uncertainty (Fig. 15).

We did not explicitly consider uncertainty in the representation of topography (Mergili et al. 2018a; Zhao and Kowalski 2020), or uncertainties in model output (which could be introduced through the $p\left({\varvec{E}}|\boldsymbol{ }{\varvec{b}}\right)$ term in Eq. [3]. As can be seen in Figs. 10 and 14, the modelled impact is underpredicted in some areas of the source zone and path. Practitioners applying this method for a risk assessment may choose to select a buffer zone around the model predictions based on professional judgement to mitigate this. Alternatively, a statistical distribution could be fitted to the distance between the observed deposit and the best-fit simulated deposit to quantify the variation in the underpredictions. This would allow the calculation of a probabilistic buffer around the modelled deposit. This could be refined by considering systematic trends in areas that are underestimated if they can be defined.

This analysis has focused on the uncertainty in the rheological parameters used in the model, which is consistent with the approach taken by other methods for forecasting flow-like landslide motion (Sun et al. 2020, 2021). However, there are some differences between the presented method and other, recent methods. The first has to do with the selection of parameter combinations for probabilistic forward analysis. In the present work, parameters are selected at regular intervals, which requires the increment between parameter values to be small enough to adequately represent the input posterior parameter distribution. This could be a strong limitation if other sources of uncertainty are considered, such as multiple uncertain path materials, as computational complexity may become an issue. In this case, more efficient methods may be required to derive runout probabilities (Calvo and Savi 2009; Sun et al. 2020, 2021).

Another difference between our method and those previously presented is the procedure for specifying the probability distributions of the input parameters. In the present work, we empirically derive the distribution based on a large database of calibrated case histories, using a method that accounts for parameter non-uniqueness and correlations. Previous work has generally assumed a probability distribution (often a normal distribution) based on expert judgement and/or small amounts of available laboratory tests (Calvo and Savi 2009; Quan Luna 2012; Sun et al. 2020). The assumption of normally distributed input parameters can ignore the parameter correlations which are present on Fig. 5 and in the Supplementary Information.

A key advantage of the presented approach is that a practitioner must only define the model geometry, the spatial distributions of rheologies, and case history weights. All other steps are then automatically executed using Matlab scripts. Thus, there is minimal additional work required by the user to apply our method, as compared to deterministic methods often used in practice today. It should be noted that this ease of use does not obviate the need for expert judgement when selecting case history weights, and interpreting the simulation results. The computation time of applying our method can be long (on the order of 10’s of hours); however, this may be reduced in the future through implementing the models on modern graphical processing units, and/or developing emulation techniques (e.g., Zhao et al. 2020).

A limitation of the prediction framework proposed in this paper is that it does not provide guidance regarding the prediction of the extent of other hazards often associated with rock avalanches, such as rock avalanche-generated sediment mass flows and air blasts (e.g., Mitchell et al. 2020a; Penna et al. 2020). These associated hazards represent an important component of the risk associated with rock avalanches. For example, at Goldau, a mud wave radiated out around the main deposit and triggered a tsunami in Lake Lauerz (Bussmann and Anselmetti 2010). Future work should incorporate a method to predict the impact area of associated hazards into the present probabilistic forecasting framework.

## Conclusions

A database of thirty-one rock avalanche case histories has been compiled, and a probabilistic rock avalanche runout methodology, which accounts for both calibration and mechanistic uncertainties, has been developed based on calibration back-analyses of the cases using Dan3D or Dan3D-Flex. It was found that forecasts are governed by both calibration and mechanistic uncertainties associated with the bulk basal resistance experienced by the rock avalanche along the path. Thus, it is recommended that a parametric or Monte-Carlo analysis, based on a probability density function derived by combining the results of multiple back-analyses, be used to forecast runout. Results from two case histories used to test the proposed framework show that the numerical model predictions can be used to refine those made by empirical techniques, and that the inclusion of expert judgement can refine the predictions further.

## Supplementary Information

Below is the link to the electronic supplementary material.

Supplementary file1 (PDF 1234 KB)
