# Supplementary material for: Probabilistic prediction of rock avalanche runout using a numerical model
Source: Landslides. 2022 Aug 15;19(12):2853–69. doi: 10.1007/s10346-022-01939-y (PMC9630252; doi:10.1007/s10346-022-01939-y)
Supplement: Supplementary file 1 — Supplementary file1 (PDF 1234 KB) [file 10346_2022_1939_MOESM1_ESM.pdf]

Probabilistic Prediction of Rock Avalanche Runout using a Numerical Model

Jordan Aaron[1,2], Scott McDougall[3], Julia Kowalski[4,5], Andrew Mitchell[3,7], Natalia Nolde[6]

[1]Geological Institute, ETH Zürich, Switzerland

[2]Now at Swiss Federal Institute for Forest, Snow and Landscape Research WSL, Switzerland

[3]Geological Engineering, The University of British Columbia, Vancouver, Canada

[4]University of Göttingen, Geoscience Center, Computational Geoscience, Göttingen, Germany

[5]Methods for Model-based Development in Computational Engineering, RWTH Aachen, Aachen, Germany

[6] Department of Statistics, The University of British Columbia, Vancouver, Canada

[7] BGC Engineering Inc., Vancouver, Canada

CASE HISTORIES AND PATH MATERIAL

The qualitative rating system used to rate the degree of confidence in the path material is given in Supplementary Table 1. The model set-up, path material and simulation constraints of the 31 case histories analysed in the present work are given in Supplementary Table 2.

Supplementary Table 1: Qualitative assessment system used to assess confidence in the path material encountered by the rock avalanche.

Rating	*Description*
High Confidence	•Pre-event images showing the presence of bedrock or glacial ice<br>•Documented evidence of entrainment, where available<br>•Documented evidence of a splash zone, where available<br>•Availability of pre-failure climatic conditions, where available
Medium Confidence	•Pre-historic or poorly documented recent cases<br>•Path material assessed based on site geomorphology<br>•Field evidence of deposit stratigraphy (where available)
Low Confidence	•Pre-historic cases<br>•Path material assessed based on site geomorphology

Supplementary Table 2: Summary of the 31 back-analysed rock avalanche case histories. Modified after the supplementary information provided in Aaron & McDougall (2019).

Name	*Setup*	*Constraints*	*Path Materials*	*Confidence*	*Select References*
1. Zymoetz	•Two rheologies used with transition selected to account for plowing of snow<br>•Frictional rheology in the source zone<br>•Voellmy rheology along the path	•Impact area<br>•Velocity<br>•Deposit distribution	•Snow<br>•Saturated sediments	High	•(Schwab et al., 2003; Boultbee et al., 2006; McDougall et al., 2006)
2. Crammont	•Two rheologies used with the transition selected to account for plowing of snow cover<br>•Frictional in source area<br>•Voellmy along path	•Impact area<br>•Deposit distribution	•Snow	High	•(Deline et al., 2011)
3. Six des Eaux Froides	•Two rheologies used, with the transition selected at the toe of the slope<br>•Frictional rheology used in the source zone<br>•Voellmy rheology used at toe of the slope<br>•Used flexible block model	•Impact area	•Saturated sediments	High	•(CREALP, 2001; Pirulli, 2005; McDougall & Hungr, 2006)

4. Huascaran	•Entrainment used<br>•Same rheology used in source zone and path	•Impact area<br>•Deposit distribution<br>•Velocity	•Glacier ice<br>•Loose, saturated substrate	High	•(Plafker & Ericksen, 1978; Chalindar, 2005; Evans et al., 2009a)
5. Kolka	•Single Voellmy Rheology used (not re-analysed in the present work)	•Impact area	•Glacial Ice	High	•(Huggel et al., 2005; McDougall, 2006; Evans et al., 2009b)
6. Mt. Meager	•One Voellmy rheology used for source zone and path	•Impact area<br>•Velocity<br>•Deposit distribution	•Loose, saturated sediments	High	•(Guthrie et al., 2012; Moretti et al., 2015; McDougall, 2017)
7. Mt. Steele	•One Voellmy rheology used for source zone and path	•Impact area	•Glacier ice	High	•(Lipovsky et al., 2008)
8. Nomash	•Entrainment used<br>•Two rheologies used, with the transition selected to correspond with zone where entrainment begins<br>•Frictional rheology used in the source zone<br>•Voellmy rheology used at the onset of entrainment	•Impact area<br>•Entrained volume	•Loose, saturated sediments	High	•(McDougall & Hungr, 2005; McDougall, 2006)
9. Sherman	•Single Voellmy Rheology used (not re-analysed in the present work)	•Impact area<br>•Deposit Thickness<br>•Velocity	•Glacier Ice	High	•(McSaveney, 1978; Sosio et al., 2012)
10. Thurweisser	•Single Voellmy Rheology used (not re-analysed in the present work)	•Impact area<br>•Velocity<br>•Deposit distribution	•Bedrock<br>•Glacier Ice	High	•(Poisel et al., 2008; Sosio et al., 2008; Favreau et al., 2010)
11. McAuley	•Two rheologies used with the transition selected downstream of the source zone<br>•Frictional rheology in the source zone<br>•Voellmy rheology along the path	•Impact area<br>•Deposit distribution	•Saturated sediments	High	•(McDougall, 2006; Brideau et al., 2012a)
12. Val Pola	•Two rheologies used, with the transition implemented at the toe of the slope<br>•Frictional rheology in the source zone<br>•Voellmy rheology along the path	•Impact area	•Saturated sediments	High	•(Crosta et al., 2004; McDougall, 2006; Pirulli & Mangeney, 2007)
13. Avalanche Lake	•Two rheologies used with transition selected to correspond where the mass overrode valley fill sediments<br>•Frictional rheology in the source zone<br>•Voellmy rheology along the path	•Impact area<br>•Deposit distribution	•Saturated sediments	Medium	•(Evans et al., 1994; Aaron & Hungr, 2016a)
14. Goldau	•Two rheologies used with the transition selected at the toe of the slope<br>•Frictional rheology in the source zone<br>•Voellmy rheology along the path<br>•Used flexible block model	•Impact area<br>•Deposit distribution	•Bedrock<br>•Saturated sediments	High	•(Berner, 2004; Thuro et al., 2006; Fitze, 2010; Aaron & Hungr, 2016b)
15. Mystery Creek	•Two rheologies used with the transition selected at the toe of the slope<br>•Frictional rheology in the source zone<br>•Voellmy rheology in the path<br>•Used flexible block model	•Impact area	•Saturated sediments	Medium	•(Eisbacher, 1983; Evans & Savigny, 1994; Nichol et al., 2002)
16. Turnoff Creek	•Two rheologies used with the transition selected at the toe of the slope<br>•Frictional rheology in the source zone<br>•Voellmy rheology in the path<br>•Used flexible block model	•Impact area<br>•Deposit distribution	•Saturated sediments	Medium	•(Beguería et al., 2009)
17. Madison Canyon	•Two rheologies used with the transition selected when the mass moves from bedrock to path sediments<br>•frictional in the source zone<br>•Voellmy on the valley floor<br>•Used flexible block model	•Impact area<br>•Deposit distribution	•Unsaturated, coarse grained sediments	High	•(Hadley, 1978; Wolter et al., 2016)
18. Chisca	•Two rheologies used with the transition selected when the mass moves from bedrock to path sediments<br>•frictional in the source zone<br>•Voellmy on the valley floor<br>•Used flexible block model	•Impact area<br>•Deposit distribution	•Bedrock<br>•Saturated Organics	•High	•(Geertsema et al., 2006)
19. Hope	•Two rheologies used with the transition selected when the mass moves from bedrock to path sediments<br>•frictional in the source zone<br>•Voellmy on the valley floor<br>•Used flexible block model	•Impact area	•Bedrock<br>•Saturated sediments	•High	•(Mathews & McTaggart, 1978; Hungr & Evans, 1996)
20. West Salt Creek	•Single Bingham rheology used for entire runout path	•Impact area<br>•Deposit distribution<br>•Velocity	•Saturated fine grained sediments	•High	•(White et al., 2015; Coe et al., 2016; Aaron et al., 2017)
21. Frank	•Two rheologies used with the transition selected at the toe of the slope<br>•Frictional rheology used in the source zone<br>•Voellmy rheology used along the path	•Impact area<br>•Deposit distribution	•Saturated sediments	High	•(Cruden & Krahn, 1978; Cruden & Hungr, 1986; McDougall, 2006)
22. Guinsaugon	•Three rheologies used with the transition selected to correspond to where the mass interacted with various path materials<br>•Frictional rheology in the source zone<br>•Two Voellmy rheologies along the path	•Impact area<br>•Deposit distribution	•Loose saturated sediments<br>•Flooded paddy field	High	•(Evans et al., 2007; Catane et al., 2008; Guthrie et al., 2009)

	•Used flexible block model				
23. Bingham Canyon	•Two rheologies used, with the transition slelected where the mass vacates the source zone<br>•Frictional rheology used in the source zone<br>•Voellmy rheology used along the path<br>•Used flexible block model	•Impact area<br>•Velocity<br>•Deposit distribution	•Bedrock	High	•(Aaron et al., 2017; Moore et al., 2017)
24. Sentinel	•Single Voellmy rheology used for both source and path<br>•Used flexible block model	•Impact area	•Unsaturated sediment	Medium	•(Castleton et al., 2016)
25. Daubensee	•Single frictional rheology used for both source and path<br>•Used flexible block model	•Impact area	•Bedrock	Medium	•(Grämiger et al., 2016)
26. Rinderhorn	•Two rheologies used with transition selected in area where the mass overrode valley sediments<br>•Frictional rheology in the source zone<br>•Voellmy rheology along the path<br>•Used flexible block model	•Impact area	•Saturated sediments	Medium	•(Grämiger et al., 2016)
27. Rautispitz	•Two rheologies used with the transition selected at the toe of the slope<br>•Used flexible block model	•Impact area	•Saturated sediment	Low	•(Nagelisen et al., 2015; Aaron et al., 2017)
28. Platten	•Two rheologies used with the transition selected at the toe of the slope<br>•Used flexible block model	•Impact area	•Saturated sediment	Low	•(Nagelisen et al., 2015)
29. Chehalis	•Single Voellmy Rheology used (not re-analysed in the present work)	•Impact area<br>•Deposit distribution	•Bedrock (above Lake)	High	•(Brideau et al., 2012b; Roberts et al., 2013; Si et al., 2018)
30. Flims	•Single Voellmy Rheology used (not re-analysed in the present work)	• Impact area	•Saturated Substrate	High	•(Pollet et al., 2005; von Poschinger & Kippel, 2009; Aaron et al., 2020)
31. Molveno	•Single Voellmy Rheology used (not re-analysed in the present work)	•Impact area	•Unsaturated Substrate	Low	•(von Wartburg et al., 2020)

SIMULATION RESULTS

The posterior probability distributions of the 22 cases back-analysed with the Voellmy rheology are shown in Figures 1 to 4 below. Plots of the maximum likelihood simulation results are presented in the supplementary information of Aaron & McDougall (2019), Aaron et al., (2020), von Wartburg et al., (2020) as well as (Evans et al., 2009b; Sosio et al., 2012; Si et al., 2018).

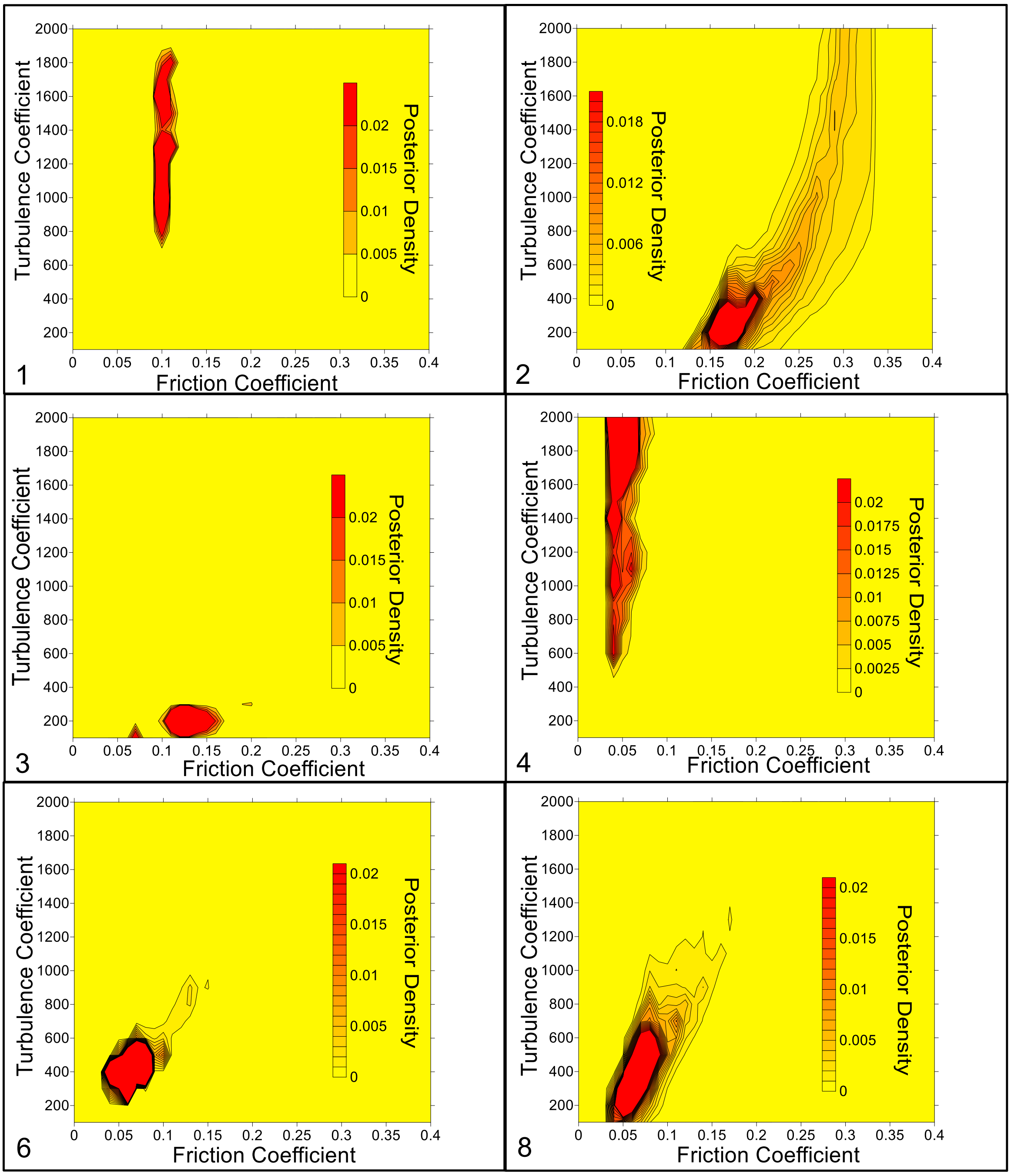

Supplementary Figure 1: Simulation results for cases 1 to 4, 6, 8. The contour intervals are as follows. 1: 0.005, 2: 0.001, 3: 0.005, 4: 0.0025, 6: 0.001, 8: 0.001.

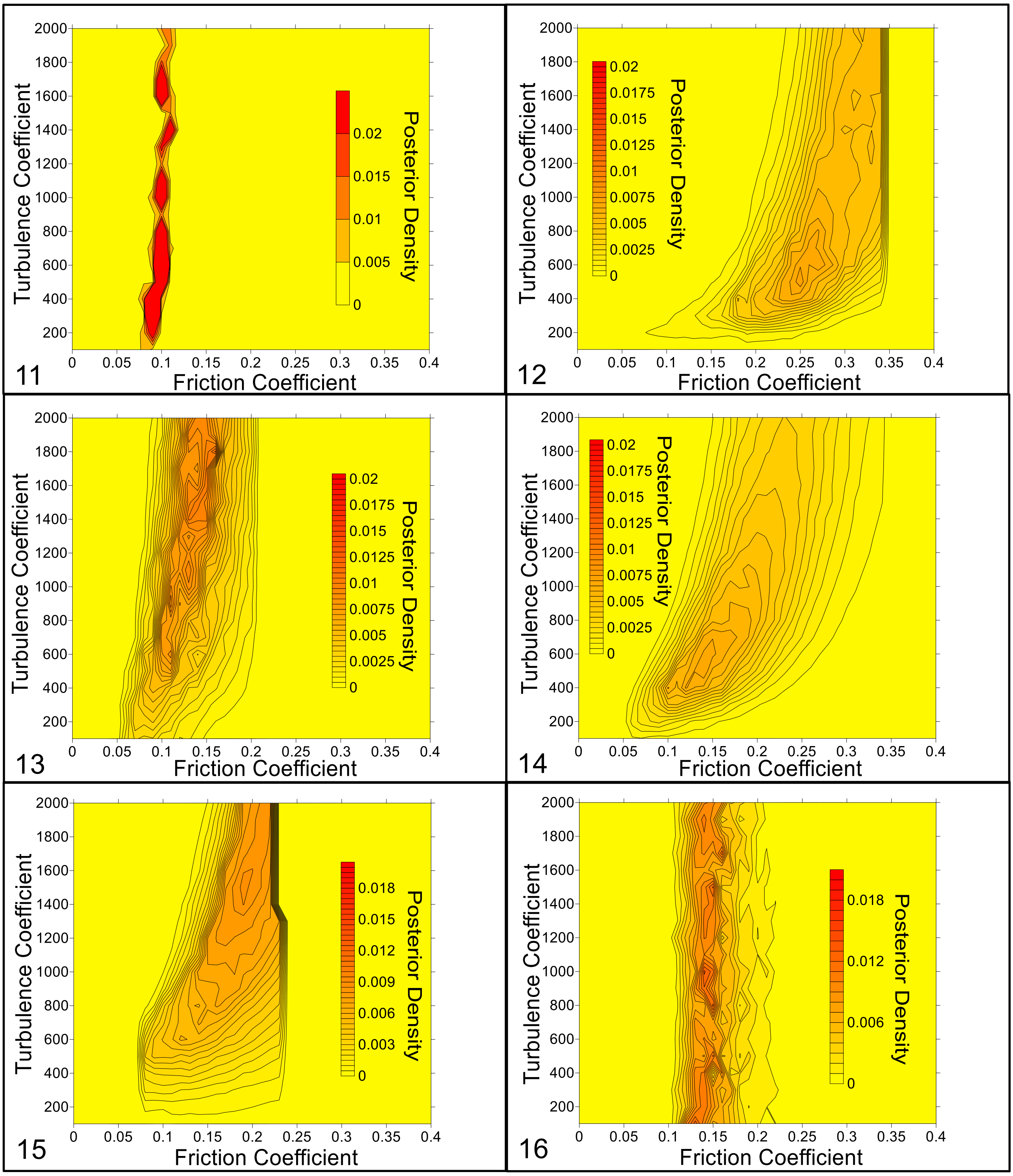

Supplementary Figure 2: Simulation results for cases 11 to 16. The contour intervals are 11: 0.005, 12: 0.0005, 13: 0.0005, 14: 0.0005, 15: 0.0005, 16: 0.001

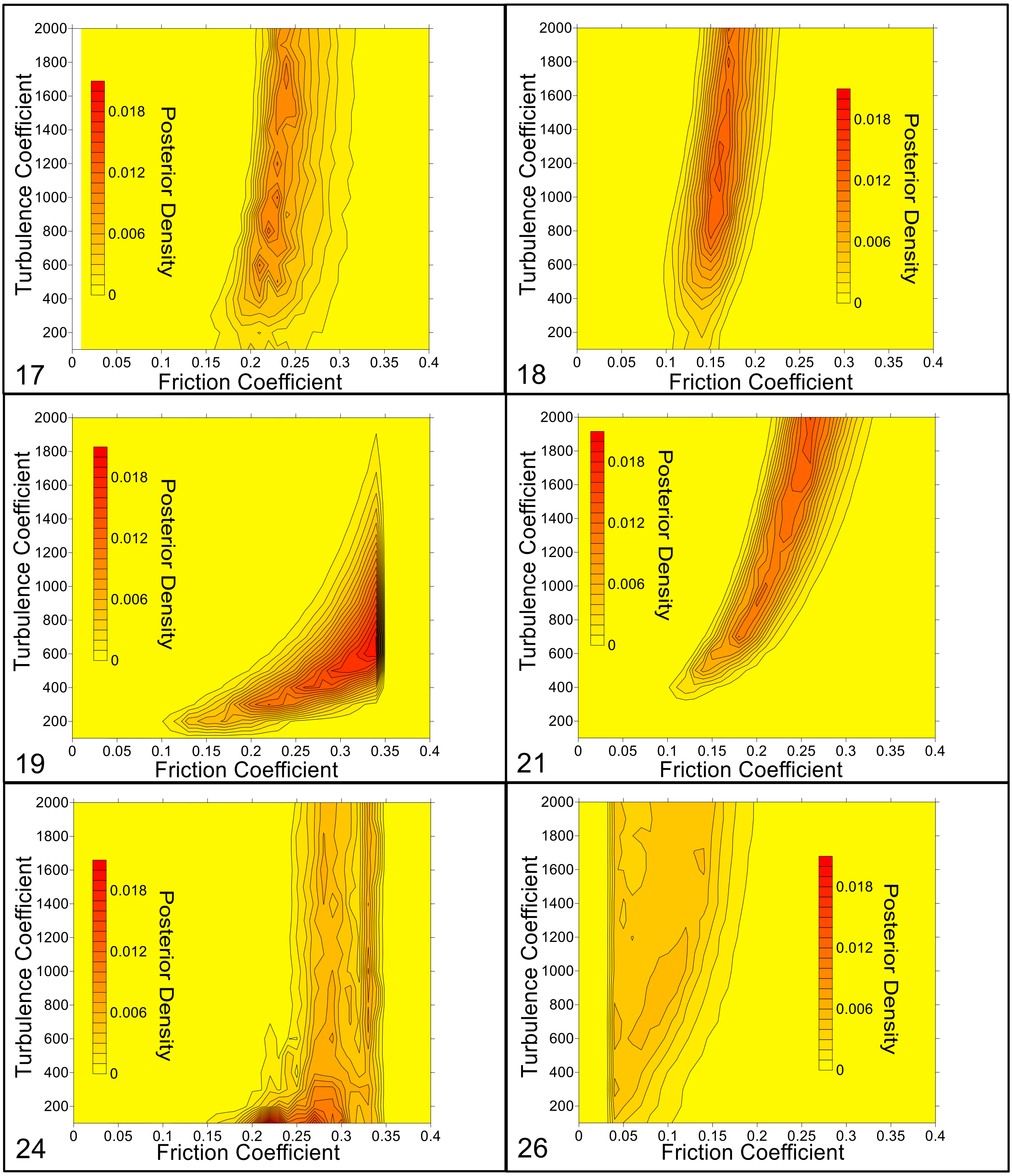

Supplementary Figure 3: Simulation results for cases 17, 18, 19, 21, 24 and 26. The contour interval for all plots is 0.001.

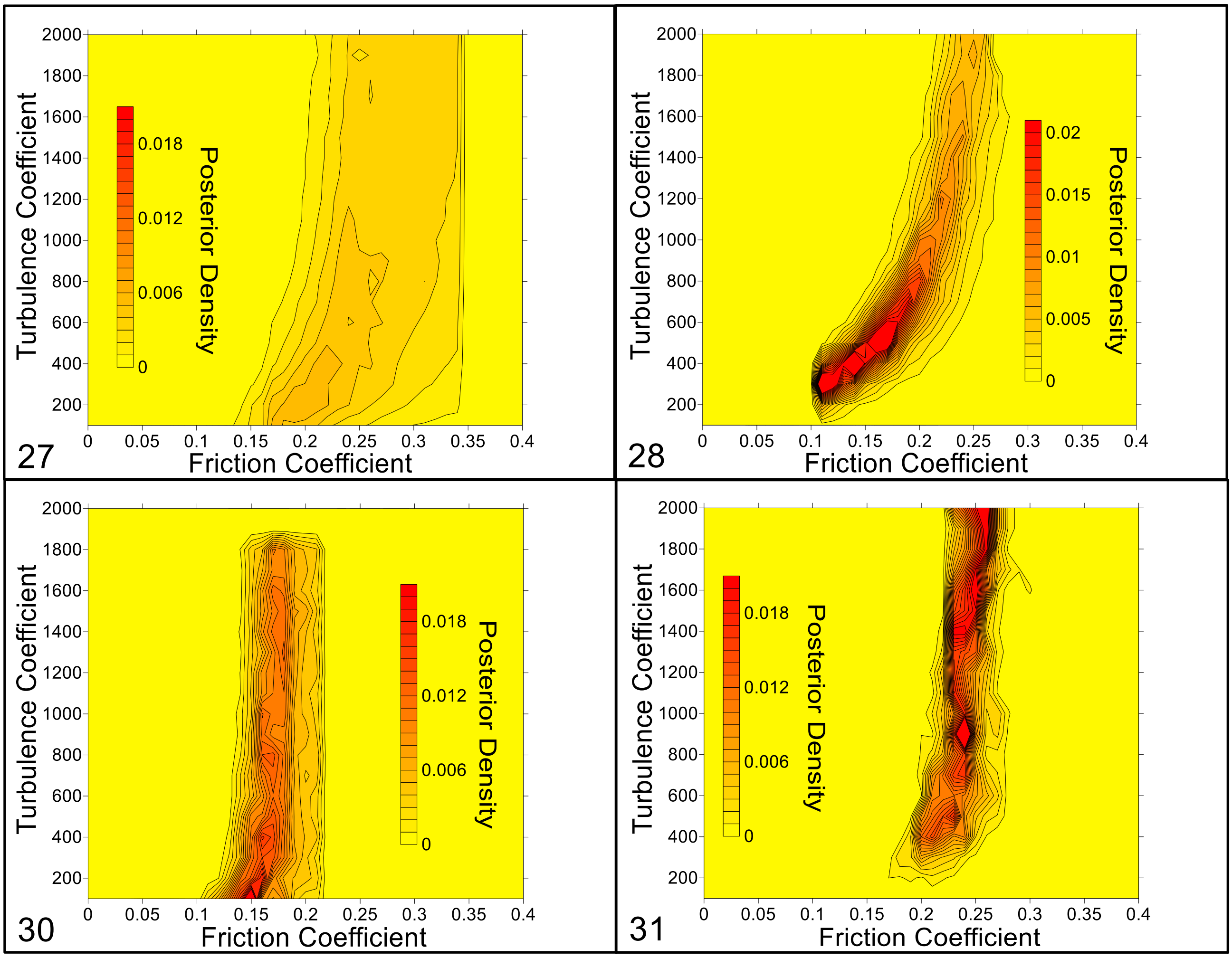

Supplementary Figure 4: Simulation results for cases 27 to 31. The contour interval for all plots is 0.001.

SUPPLEMENTARY REFERENCES

Aaron, J., & Hungr, O. (2016a). Dynamic analysis of an extraordinarily mobile rock avalanche in the Northwest Territories, Canada. *Canadian Geotechnical Journal*, *53*(6), 899–908. https://doi.org/10.1139/cgj-2015-0371

Aaron, J., & Hungr, O. (2016b). Dynamic simulation of the motion of partially-coherent landslides. *Engineering Geology*, *205*, 1–11. https://doi.org/10.1016/j.enggeo.2016.02.006

Aaron, J., McDougall, S., Moore, J. R., Coe, J. A., & Hungr, O. (2017). The role of initial coherence and path materials in the dynamics of three rock avalanche case histories. *Geoenvironmental Disasters*, *4*, 5. https://doi.org/10.1186/s40677-017-0070-4

Aaron, J., Wolter, A., Loew, S., & Volken, S. (2020). Understanding Failure and Runout Mechanisms of the Flims Rockslide/Rock Avalanche. *Frontiers in Earth Science*, *8*(June), 1–19. https://doi.org/10.3389/feart.2020.00224

Beguería, S., van Hees, M. J., & Geertsema, M. (2009). Comparison of three landslide runout models on the Turnoff Creek rock avalanche, British Columbia. In *Landslide Processes Conference: A Tribute to Theo von Asch* (pp. 243–247). Strasburg.

Berner, C. (2004). *Der Bergsturz von Goldau, Diploma Thesis*. ETH, Zurich.

Boultbee, N., Stead, D., Schwab, J., & Geertsema, M. (2006). The Zymoetz River rock avalanche, June 2002, British Columbia, Canada. *Engineering Geology*, *83*(1–3), 76–93. https://doi.org/10.1016/j.enggeo.2005.06.038

Brideau, M.-A., McDougall, S., Stead, D., Evans, S. G., Couture, R., & Turner, K. (2012a). Three-dimensional distinct element modelling and dynamic runout analysis of a landslide in gneissic rock, British Columbia, Canada. *Bulletin of Engineering Geology and the Environment*, *71*, 467–486. https://doi.org/10.1007/s10064-012-0417-8

Brideau, M. A., Sturzenegger, M., Stead, D., Jaboyedoff, M., Lawrence, M., Roberts, N., Ward, B., Millard, T., & Clague, J. (2012b). Stability analysis of the 2007 Chehalis lake landslide based on long-range terrestrial photogrammetry and airborne LiDAR data. *Landslides*, *9*(1), 75–91. https://doi.org/10.1007/s10346-011-0286-4

Castleton, J. J., Moore, J. R., Aaron, J., Christl, M., & Ivy-Ochs, S. (2016). Dynamics and legacy of 4.8 ka rock avalanche that dammed Zion Canyon, Utah, USA. *GSA Today*, *26*(6), 4–9. https://doi.org/10.1130/GSATG269A.1

Catane, S. G., Cabria, H. B., Zarco, M. A. H., Saturay, R. M., & Mirasol-Robert, A. A. (2008). The 17 February 2006 Guinsaugon rock slide-debris avalanche, Southern Leyte, Philippines: Deposit characteristics and failure mechanism. *Bulletin of Engineering Geology and the Environment*, *67*, 305–320. https://doi.org/10.1007/s10064-008-0120-y

Chalindar, S. (2005). *Modélisation d ’ avalanches rocheuses, Internship Report*. Civil and Environmental Engineering (ENAC) - Laboratory for Rock Mechanics (LMR).

Coe, J. A., Baum, R. L., Allstadt, K. E., Kochevar, B. F., Schmitt, R. G., Morgan, M. L., White, J. L., Stratton, B. T., Hayashi, T. A., & Kean, J. W. (2016). Rock-avalanche dynamics revealed by large-scale field mapping and seismic signals at a highly mobile avalanche in the West Salt Creek valley, western Colorado. *Geosphere*, *12*(2), 607–631. https://doi.org/10.1130/GES01265.1

CREALP, C. de R. sur L. A. (2001). *Eboulement du 20 Mai du Six des Eaux Froides pres du Rawyl (Valais)*. Sion, Switzerland.

Crosta, G. B., Chen, H., & Lee, C. F. (2004). Replay of the 1987 Val Pola Landslide, Italian Alps. *Geomorphology*, *60*(1–2), 127–146. https://doi.org/10.1016/j.geomorph.2003.07.015

Cruden, D. ., & Krahn, J. (1978). Frank Rockslide, Alberta, Canada. In B. Voight (Ed.), *Rockslides and Avalanches, Vol 1 Natural Phenomena* (pp. 97–112). Amsterdam: Elsevier Scientific Publishing.

Cruden, D., & Hungr, O. (1986). The debris of the Frank Slide and theories of rockslide-avalanche mobility. *Canadian Journal of Earth Sciences*, *23*(3), 425–432. https://doi.org/https://doi.org/10.1139/e86-044

Deline, P., Alberto, W., Broccolato, M., Hungr, O., Noetzli, J., Ravanel, L., & Tamburini, A. (2011). The December 2008 Crammont rock avalanche, Mont Blanc massif area, Italy. *Natural Hazards and Earth System Science*, *11*(12), 3307–3318. https://doi.org/10.5194/nhess-11-3307-2011

Eisbacher, G. . (1983). *Field Trip Guidebook - Slope Stability and Mountain Torrents Fraser Lowlands and Southern Coast Mountains, British Columbia*.

Evans, S. G., Bishop, N. F., Fidel Smoll, L., Valderrama Murillo, P., Delaney, K. B., & Oliver-Smith, A. (2009a). A re-examination of the mechanism and human impact of catastrophic mass flows originating on Nevado Huascaran, Cordillera Blanca, Peru in 1962 and 1970. *Engineering Geology*, *108*(1–2), 96–118. https://doi.org/10.1016/j.enggeo.2009.06.020

Evans, S. G., Guthrie, R. H., Roberts, N. J., & Bishop, N. F. (2007). The disastrous 17 February 2006 rockslide-debris avalanche on Leyte Island, Philippines: a catastrophic landslide in tropical mountain terrain. *Natural Hazards and Earth System Science*, *7*, 89–101. https://doi.org/10.5194/nhess-7-89-2007

Evans, S. G., Tutubalina, O. V., Drobyshev, V. N., Chernomorets, S. S., McDougall, S., Petrakov, D. A., & Hungr, O. (2009b). Catastrophic detachment and high-velocity long-runout flow of Kolka Glacier, Caucasus Mountains, Russia in 2002. *Geomorphology*, *105*(3–4), 314–321. https://doi.org/10.1016/j.geomorph.2008.10.008

Evans, S., Hungr, O., & Enegren, E. . (1994). The Avalanche Lake rock avalanche, Mackenzie Mountains, Northwest Territories, Canada: description, dating and dynamics. *Canadian Geotechnical Journal*, *31*(5), 749–768. https://doi.org/https://doi.org/10.1139/t94-086

Evans, S., & Savigny, K. (1994). Landslides in the Vancouver–Fraser Valley–Whistler region. In J. W. . Monger (Ed.), *Geology and Geological Hazards of the Vancouver Region, southwestern British Columbia* (pp. 251–286). Geological Survey of Canada, Bulletin 481.

Favreau, P., Mangeney, A., Lucas, A., Crosta, G., & Bouchut, F. (2010). Numerical modeling of landquakes. *Geophysical Research Letters*, *37*(15). https://doi.org/10.1029/2010GL043512

Fitze, P. (2010). *Runout analysis of rapid , flow-like landslides, Master's Thesis*. Hochschule für Technik Rapperswil.

Geertsema, M., Clague, J. J., Schwab, J. W., & Evans, S. G. (2006). An overview of recent large catastrophic landslides in northern British Columbia, Canada. *Engineering Geology*, *83*(1–3), 120–143. https://doi.org/10.1016/j.enggeo.2005.06.028

Grämiger, L. M., Moore, J. R., Vockenhuber, C., Aaron, J., Hajdas, I., & Ivy-Ochs, S. (2016). Two early Holocene rock avalanches in the Bernese Alps (Rinderhorn, Switzerland). *Geomorphology*, *268*, 207–221. https://doi.org/10.1016/j.geomorph.2016.06.008

Guthrie, R. H., Evans, S. G., Catane, S. G., Zarco, M. A. H., & Saturay, R. M. (2009). The 17 February 2006 rock slide-debris avalanche at Guinsaugon Philippines: A synthesis. *Bulletin of Engineering Geology and the Environment*, *68*(2), 201–213. https://doi.org/10.1007/s10064-009-0205-2

Guthrie, R. H., Friele, P., Allstadt, K., Roberts, N., Evans, S. G., Delaney, K. B., Roche, D., Clague, J. J., & Jakob, M. (2012). The 6 August 2010 Mount Meager rock slide-debris flow, Coast Mountains, British Columbia: characteristics, dynamics, and implications for hazard and risk assessment. *Natural Hazards and Earth System Science*, *12*, 1277–1294. https://doi.org/10.5194/nhess-12-1277-2012

Hadley, J. (1978). Madison Canyon rockslide, Montana, USA. In B. Voight (Ed.), *Rockslides and Avalanches, Vol 1 Natural Phenomena1* (pp. 172–180). Elsevier Scientific Publishing.

Huggel, C., Zgraggen-Oswald, S., Haeberli, W., Kääb, A., Polkvoj, A., Galushkin, I., & Evans, S. G. (2005). The 2002 rock/ice avalanche at Kolka/Karmadon, Russian Caucasus: assessment of extraordinary avalanche formation and mobility, and application of QuickBird satellite imagery. *Natural Hazards and Earth System Science*, *5*, 173–187. https://doi.org/10.5194/nhess-5-173-2005

Hungr, O., & Evans, S. (1996). Rock Avalanche Runout Prediction Using a Dynamic Model. In K. Senneset (Ed.), *Procs., 7th. International Symposium on Landslides* (pp. 233–238). Trondheim, Norway: A. A. Balkema.

Lipovsky, P. S., Evans, S. G., Clague, J. J., Hopkinson, C., Couture, R., Bobrowsky, P., Ekström, G., Demuth, M. N., Delaney, K. B., Roberts, N. J., Clarke, G., & Schaeffer, A. (2008). The July 2007 rock and ice avalanches at Mount Steele, St. Elias Mountains, Yukon, Canada. *Landslides*, *5*(4), 445–455. https://doi.org/10.1007/s10346-008-0133-4

Mathews, W. H., & McTaggart. (1978). Hope Rockslides, British Columbia, Canada. In B. Voight (Ed.), *Rockslides and Avalanches, Vol 1 Natural Phenomena* (pp. 259–275). New York: Elsevier.

McDougall, S. (2006). *A New Continuum Dynamic Model For the Analysis of Extremely Rapid Landslide Motion Across Complex 3D Terrain, PhD Thesis*. University of British Columbia.

McDougall, S. (2017). 2014 Canadian Geotechnical Colloquium: Landslide runout analysis — current practice and challenges, *54*(5), 605–620.

McDougall, S., Boultbee, N., Hungr, O., Stead, D., & Schwab, J. W. (2006). The Zymoetz River landslide, British Columbia, Canada: description and dynamic analysis of a rock slide–debris flow. *Landslides*, *3*(3), 195–204. https://doi.org/10.1007/s10346-006-0042-3

McDougall, S., & Hungr, O. (2005). Dynamic modelling of entrainment in rapid landslides. *Canadian Geotechnical Journal*, *42*(5), 1437–1448. https://doi.org/10.1139/t05-064

McDougall, S., & Hungr, O. (2006). Landslide Dynamic Analysis in 2D and 3D. In *Sea to Sky Geotechnique, the 59th Canadian Geotechnical Conference*.

McSaveney, M. J. (1978). Sherman glacier rock avalanche, alaska, U.S.A. In *Developments in Geotechnical Engineering* (Vol. 14, pp. 197–258). Elsevier Scientific Publishing Company. https://doi.org/10.1016/B978-0-444-41507-3.50014-3

Moore, J. R., Pankow, K. L., Ford, S. R., Koper, K. D., Hale, J. M., Aaron, J., & Larsen, C. F. (2017). Dynamics of the Bingham Canyon rock avalanches (Utah, USA) resolved from topographic, seismic, and infrasound data. *Journal of Geophysical Research: Earth Surface*, *122*(3), 615–640. https://doi.org/10.1002/2016JF004036

Moretti, L., Allstadt, K., Mangeney, A., Capdeville, Y., Stutzmann, E., & Bouchut, F. (2015). Numerical modeling of the Mount Meager landslide constrained by its force history derived from seismic data. *Journal of Geophysical Research B: Solid Earth*, *120*(4), 2579–2599. https://doi.org/10.1002/2014JB011426

Nagelisen, J., Moore, J. R., Vockenhuber, C., & Ivy-Ochs, S. (2015). Post-glacial rock avalanches in the Obersee Valley, Glarner Alps, Switzerland. *Geomorphology*, *238*, 94–111. https://doi.org/10.1016/j.geomorph.2015.02.031

Nichol, S., Hungr, O., & Evans, S. (2002). Large-scale brittle and ductile toppling of rock slopes. *Canadian Geotechnical Journal*, *39*(4), 773–788. https://doi.org/10.1139/T02-027

Pirulli, M. (2005). *Numerical Modelling of Landslide Runout, PhD Thesis*. Politecnico Di Torino.

Pirulli, M., & Mangeney, A. (2007). Results of Back-Analysis of the Propagation of Rock Avalanches as a Function of the Assumed Rheology. *Rock Mechanics and Rock Engineering*, *41*(1), 59–84. https://doi.org/10.1007/s00603-007-0143-x

Plafker, G., & Ericksen, G. . (1978). Nevados Huascaran avalanches, Peru. In B. Voight (Ed.), *Rockslides and Avalanches, Vol 1 Natural Phenomena* (pp. 277–314). Amsterdam: Elsevier Scientific Publishing.

Poisel, R., Preh, A., & Hungr, O. (2008). Run Out of Landslides - Continuum Mechanics versus Discontinuum Mechanics Models. *Geomechanik Und Tunnelbau*, *1*(5), 358–366. https://doi.org/10.1002/geot.200800036

Pollet, N., Cojean, R., Couture, R., Schneider, J., Strom, A. L., Voirin, C., & Wassmer, P. (2005). A slab-on-slab model for the Flims rockslide (Swiss Alps), *600*, 587–600. https://doi.org/10.1139/T04-122

Roberts, N. J., Mckillop, R. J., Lawrence, M. S., Psutka, J. F., Clague, J. J., Brideau, M.-A., & Ward, B. C. (2013). Impacts of the 2007 Landslide-Generated Tsunami in Chehalis Lake, Canada. In M. C., C. P., & S. K. (Eds.), *Landslide Science and Practice* (Vol. 6). Springer Berlin Heidelberg. https://doi.org/https://doi.org/10.1007/978-3-642-31319-6_19

Schwab, J., Geertsema, M., & Evans, S. G. (2003). Catastrophic rock avalanches, west-central British Columbia. In *Proceedings of the 3rd Canadian conference on geotechnique and natural hazards* (pp. 252–259). Edmonton, Alberta.

Si, P., Aaron, J., McDougall, S., Lu, J., Yu, X., Roberts, N. J., & Clague, J. J. (2018). A non-hydrostatic model for the numerical study of landslide-generated waves. *Landslides*, *15*(4), 711–726. https://doi.org/10.1007/s10346-017-0891-y

Sosio, R., Crosta, G. B., Chen, J. H., & Hungr, O. (2012). Modelling rock avalanche propagation onto glaciers. *Quaternary Science Reviews*, *47*, 23–40. https://doi.org/10.1016/j.quascirev.2012.05.010

Sosio, R., Crosta, G. B., & Hungr, O. (2008). Complete dynamic modeling calibration for the Thurwieser rock avalanche (Italian Central Alps). *Engineering Geology*, *100*(1–2), 11–26. https://doi.org/10.1016/j.enggeo.2008.02.012

Thuro, K. K., Berner, C., & Eberhardt, E. (2006). Der Bergsturz von Goldau 1806 - Was wissen wir 200 Jahre nach der Katastrophe? *Bull. Angew. Geol.*, *11/2*, 13–24. https://doi.org/http://doi.org/10.5169/seals-226165

von Poschinger, A., & Kippel, T. (2009). Alluvial deposits liquefied by the Flims rock slide. *Geomorphology*, *103*(1), 50–56. https://doi.org/10.1016/j.geomorph.2007.09.016

von Wartburg, J., Ivy-Ochs, S., Aaron, J., Martin, S., Leith, K., Rigo, M., Vockenhuber, C., Campedel, P., & Viganò, A. (2020). Constraining the Age and Source Area of the Molveno landslide Deposits in the Brenta Group, Trentino Dolomites (Italy). *Frontiers in Earth Science*, *8*(June), 1–18. https://doi.org/10.3389/feart.2020.00164

White, J. L., Morgan, M. L., & Berry, K. A. (2015). *Bulletin 55 - The West Salt Creek Landslide: A Catastrophic Rockslide and Rock/Debris Avalanche in Mesa County*. *Bulletin 55*. Golden, CO: Colorado Geological Survey.

Wolter, A., Gischig, V., Stead, D., & Clague, J. J. (2016). Investigation of geomorphic and seismic effects on the 1959 Madison Canyon, Montana, landslide using an integrated field, engineering geomorphology mapping, and numerical modelling approach. *Rock Mechanics and Rock Engineering*, *49*(6), 2479–2501. https://doi.org/10.1007/s00603-015-0889-5
